# Tuned interfacial hydrogen bonds for enhanced H_2_ electrocatalysis kinetics on Ti_2_C MXenes

**DOI:** 10.1038/s42004-026-02099-z

**Published:** 2026-07-13

**Authors:** Lin Chen, Johanna Rosen, Jonas Björk

**Affiliations:** Materials Design Division, Department of Physics, Chemistry and Biology, IFM, Linköping University, Linköping, Sweden

**Keywords:** Electrocatalysis, Electrocatalysis, Reaction kinetics and dynamics

## Abstract

The kinetics of proton-coupled electron transfer (PCET) in electrocatalytic hydrogen evolution reactions (HER) are profoundly influenced by the hydrogen-bond (H-bond) network structure within the electric double layer. Here, we elucidate the role of surface terminations in modulating PCET kinetics on Ti_2_CT_2_ (T = O, OH) MXenes under acidic conditions, employing our in-house developed constant-potential constrained molecular dynamics approach. Our simulations reveal that mixed O-/OH-terminated Ti_2_C MXenes exhibit significantly enhanced HER kinetics compared to pure O-terminated surfaces. By analyzing the interfacial water structure, we demonstrate that OH terminations shorten the basal water-MXene distance and reorganize the electric double layer, transforming fragmented H-bond networks into continuous ones that facilitate efficient proton transfer. This structural arrangement facilitates hydronium ion (H_3_O^+^) dehydration while stabilizing critical reaction intermediates, particularly Zundel cations (H_5_${{{\rm{O}}}}_2^+$), thereby reducing the activation energy barriers for both the Volmer and Heyrovsky steps. Crucially, we establish a mechanistic link between H-bond connectivity and PCET kinetics – a framework extending beyond the conventional ΔG_H_ descriptor. Moreover, our methodology demonstrates how dynamic interfacial processes can be rigorously captured, paving the way for more accurate simulations of electrified interfaces.

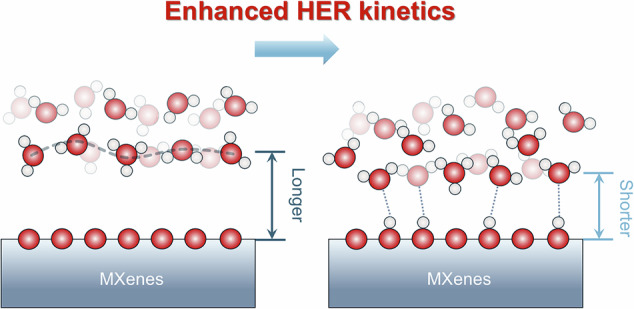

## Abstract (web summary)

The potential-dependent restructuring of hydrogen bond networks within the electric double layer may dominate kinetic bottlenecks in proton-coupled electron transfer (PCET) at the electrocatalytic interface, but this phenomenon tends to be neglected in conventional density functional theory calculations. Here, the authors use a constant-potential constrained molecular dynamics approach to study PCET kinetics on Ti_2_CT_2_ (T = O, OH) MXenes under acidic conditions, showing that hydroxyl surface terminations shorten the basal water-MXene distance, transforming fragmented hydrogen bond networks into continuous ones that facilitate efficient proton transfer.

## Introduction

The electrochemical production of hydrogen through the hydrogen evolution reaction (HER) represents a cornerstone of sustainable energy technologies^1,2^, with its kinetics being fundamentally governed by the proton-coupled electron transfer (PCET) process at the electrode-electrolyte interface^3–5^. While traditional computational catalyst design has focused primarily on optimizing the thermodynamic descriptors such as hydrogen adsorption free energy (*ΔG*_*H*_)^6–9^, recent advances have revealed that the interfacial hydrogen-bond (H-bond) networks within the electric double layer (EDL) critically influence proton transport and reaction kinetics^10–15^. This new understanding highlights the need to understand how atomic-scale interfacial structures mediate PCET kinetics—a challenge that requires precise control over both catalyst surface chemistry and the adjacent electrolyte structure.

A major challenge in electrocatalysis lies in unraveling how interfacial water structure and surface termination chemistry collectively regulate PCET kinetics^5,16^. Under operational electrode potentials, dynamic restructuring occurs at multiple levels: (1) surface structures can undergo dynamical reconfiguration, including adsorption, desorption, or atomic rearrangement; (2) interfacial water molecules reorganize into distinct H-bond networks; and (3) the fractional charge of protons in the outer Helmholtz plane fluctuates^17^, inducing potential-dependent reaction energetics. Critically, these factors are intertwined—surface terminations influences the local electric field, which in turn modulates water orientation and proton transport kinetics. This multi-component interdependence complicates the establishment of structure-activity relationships, necessitating approaches that simultaneously resolve atomic-scale surface chemistry and EDL restructuring under realistic electrode potentials.

MXenes, a class of two-dimensional transition metal carbides/nitrides^18–20^, offer an ideal template to investigate these interfacial effects due to their tunable surface terminations (e.g., -O, -OH)^21–23^. Prior studies have demonstrated that MXenes exhibit promising HER activity^24,25^, but the mechanistic role of termination groups in modulating the EDL and H-bond networks remains unclear. Conventional density functional theory (DFT) calculations typically treat the EDL as static, neglecting the potential-dependent restructuring of H-bond networks that recent experiments suggest may dominate kinetic bottlenecks. This limitation underscores the need for advanced simulation techniques that can model electrified interfaces under realistic electrochemical conditions.

In this study, we applied a hybrid computational framework that integrates a constant-potential algorithm with constrained molecular dynamics (CP-CMD) simulations to unravel how surface terminations on Ti_2_CT_2_ (T = O, OH) MXenes regulate interfacial H-bond networks and HER kinetics in acidic media. Ti_2_CT_2_ MXene was selected as the model system due to its well-established synthesis protocols^26^. The stable O-/OH-termination patterns on MXenes under electrochemical conditions were identified through a combined approach of configuration sampling and surface Pourbaix analysis. Our CP-CMD simulations reveal that mixed O/OH-terminated Ti_2_C MXenes exhibit significantly enhanced HER kinetics compared to pure O-terminated surfaces. Mixed O/OH terminations uniquely anchor interfacial water via OH groups, shortening the water-MXene distance and restructuring the EDL into a continuous H-bond network - a structural advantage absent in pure O-terminated MXenes, as evidenced by the interfacial water structure analysis. Our findings establish a mechanistic framework wherein H-bond network connectivity—distinct from traditional thermodynamic descriptors (ΔG_H_)—directly regulates PCET kinetics. This insight extends beyond MXene-based HER electrocatalysis, offering a general design principle for optimizing electrochemical interfaces through EDL microstructure control. Furthermore, the CP-CMD simulation tools provide a rigorous framework for simulating dynamic electrochemical interfaces at atomic resolution, bridging the gap between conventional static DFT models and operando experimental observations.

## Results

### Surface termination dynamics under electrocatalytic conditions

**Fig. 1:**
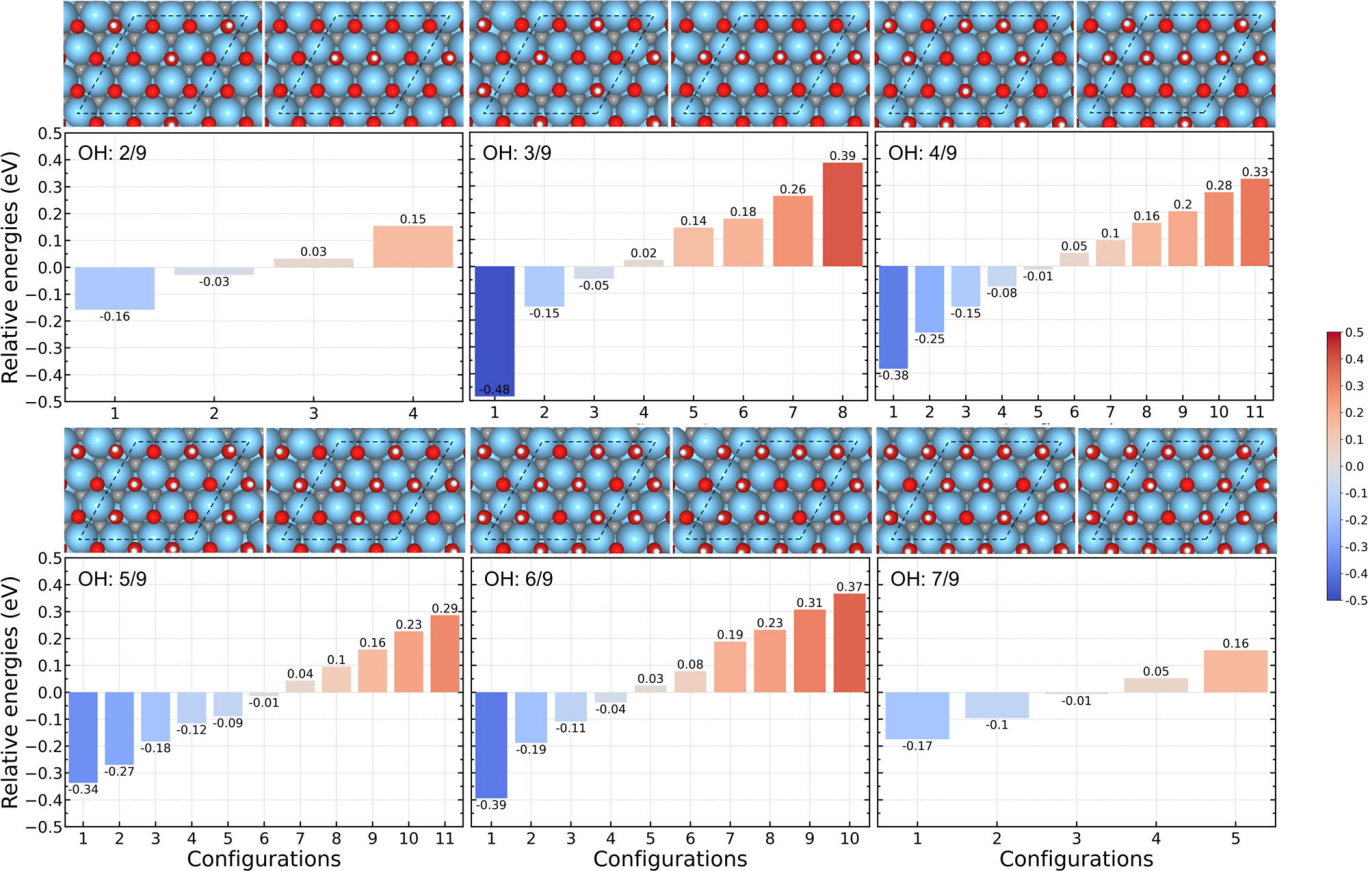
Stability distribution of various topological arrangements for mixed O-/OH- terminations on Ti_2_C MXene. The OH coverage ranges from 2/9 to 7/9. The relative energies were calculated with respect to the average energies for each OH coverage. The color bar on the right indicates relative energy values ranging from −0.5 eV (dark blue, representing the highest stability or lowest energy) to +0.5 eV (dark red, indicating the lowest stability or highest energy). The most stable configuration for each OH coverage is displayed on the left above each stability distribution plot, while the most unstable configuration is shown on the right. For each OH coverage, configurations with energy differences of less than 0.05 eV have been grouped together to simplify the stability distribution plot and enhance clarity.

The etching of monoatomic Al layers from the Ti_2_AlC MAX phase precursor using aqueous hydrogen fluoride typically yields O-, OH-, and F-terminated MXenes^19,23^. Given that F-terminated surfaces are generally considered to be inactive for H_2_ electrocatalysis^21^, this study focuses on Ti_2_C MXene with pure O- and mixed O-/OH-terminations as the primary adsorbing species. This approach is further motivated by experimental studies showing pathways for the removal of F, enabling surfaces terminated solely by O/OH^27^. The ambiguity in experimentally quantifying O-/OH-terminations on MXenes^23^ and the environmental sensitivity of these terminations^22^ motivate our systematic investigation of the thermodynamically stable configurations under relevant electrocatalytic conditions. We started with a systematic exploration of stable configurations for various distributions of O- and OH-terminations on Ti_2_C MXene surfaces. The equilibrium lattice constants *a* for pure O- and mixed O-/OH-terminated Ti_2_C MXene were calculated (Supplementary Fig. 1). The results show that the in-plane lattice constant initially contracts slightly at low OH coverage (1/9) and then expands by less than 1% overall with increasing OH coverage, reaching its maximum value for the pure OH-terminated surface. The increasing lattice constant is driven by the elongation of the Ti-O bond, which increases from 1.98 to 2.17 Å upon full hydroxylation. For each mixed O-/OH- terminated Ti_2_C MXene, with OH coverage ranging from 2/9 to 7/9, we examined all possible topological arrangements of the surface terminations species. The energies for each configuration were calculated, and the resulting stability distributions for each OH coverage are presented in Fig. 1. The impact of surface termination topologies on relative stability can be significant, for instance, the relative energy difference can reach 0.87 eV for an OH coverage of 3/9. The most stable structure for each OH coverage, depicted with the corresponding stability distribution in Fig. 1, indicates that OH groups prefer an even distribution across the surface, which enhances the stability. In contrast, clustering of OH species can destabilize the system, which agrees with previous studies^28,29^.

**Fig. 2:**
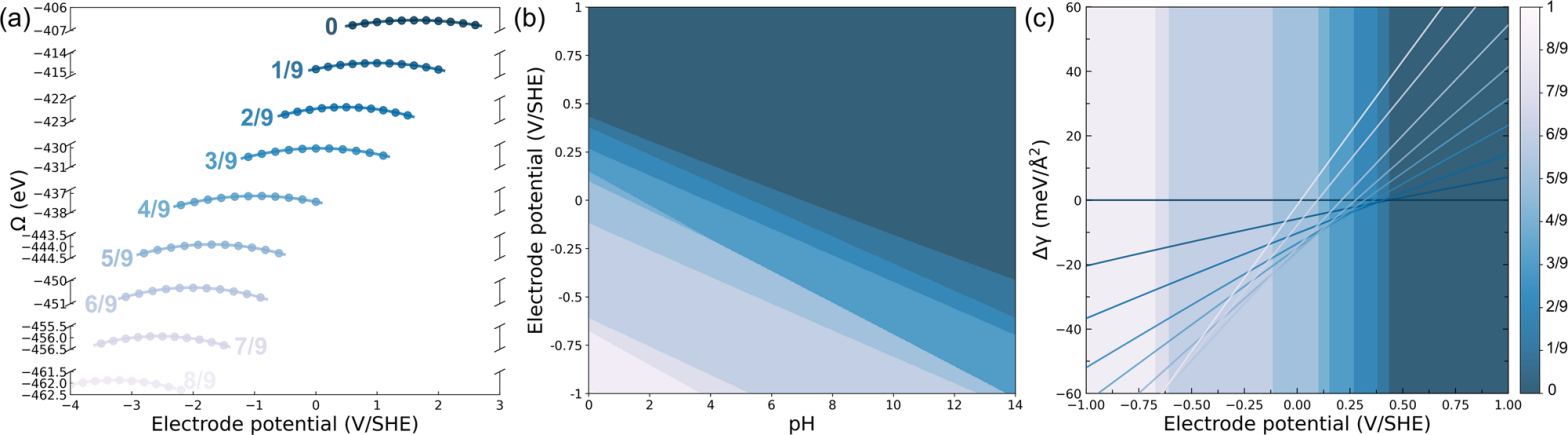
Surface Pourbaix diagram. **a** The (electronic) grand-canonical potential energy Ω of O- and mixed O-/OH- terminated Ti_2_C MXene as a function of electrode potential (V/SHE). OH coverage indicated for each system is labeled next to the corresponding fitted line; **b** Surface Pourbaix diagram, i.e., a map of the stable phases of Ti_2_C MXene with various combinations of O and OH terminations as a function of pH and electrode potential; **c** Free energy of hydrogen adsorption per surface area on oxygen-terminated Ti_2_C MXene at electrochemical interfaces as a function of electrode potential under pH = 0 conditions. The grand canonical potential energy for each considered configuration is used to construct the stability map. The color bar shows the coverage of OH termination species.

Having established the stable termination topologies for each mixed O-/OH-terminated Ti_2_C MXene, we calculated the electronic grand-canonical potential energies, Ω, for O- and mixed O-/OH-terminated Ti_2_C MXene as a function of the electrode potential (V/SHE). The electric grand-canonical potential accounts for the total energies of an electrode-electrolyte system at an applied electrode potential, allowing the number of electrons to vary. The results are presented in Fig. 2a. The fitted parameters for the quadratic relationship of the potential-dependent Ω are summarized in Supplementary Table 1.

To determine the stable equilibrium structures of Ti_2_C MXene under an electrochemical environment, we constructed a surface Pourbaix diagram that utilizes the concept of the computational hydrogen electrode (CHE)^30^ while incorporating further advancements. Specifically, the free energies of hydrogen adsorption were calculated using the potential-dependent energies (Ω) instead of the conventional total energies for the vacuum/electrode interface, which neglects the effects of the electric field on the interface stability. The capacitive charging nature of the EDL has been recognized as a critical factor that influences the thermodynamic and kinetic behavior of the electrochemistry interfaces^31–33^. The surface Pourbaix diagram for Ti_2_C MXene terminated with various combinations of O and OH species is shown in Fig. 2b. The phase diagram reveals competitive hydrogen adsorption/desorption behavior under strongly acidic conditions. As the electrode potential increases, the OH-termination coverage on Ti_2_C MXene decreases due to the lower electrochemical potential of hydrogen (the sum of the electrochemical potentials of the proton and the electron), resulting in a higher energy penalty for hydrogen adsorption at the O-terminated Ti_2_C MXene surface. It is noteworthy that the boundaries between the considered phases exhibit different slopes, indicating that they deviate from ideal Nernstian behavior. Although pH affects the relative stability of each phase only through the ${{{{k}}}}_{{{{\rm{B}}}}}{{{\rm{Tln}}}}(10)\cdot {{{\rm{pH}}}}$ term in Equation (3), the electrode potential affects the difference in the interface energies denoted by the different capacitances for each phase in Equation (6), thereby breaking the ideal Nernstian behavior^34^.

For comparison, we also constructed a surface Pourbaix diagram without considering the electric field’s effect on the stability of the interface (as shown in Supplementary Fig. 2), i.e., the energy associated with each system at the potential of zero charge (PZC) was employed to derive the free energy of hydrogen adsorption per surface area. By construction, the boundaries between each phase all exhibit a slope of 59 mV/pH, indicating an ideal Nernstian behavior^35^. Our results reveal that the interfacial energetics exhibit potential-dependent character, which may explain why the ideal Nernstian behavior has not been observed in experiments^36^. Figure 2c presents the potential-dependent free energies of hydrogen adsorption per unit surface area on O-terminated Ti_2_C MXene under acidic conditions (pH = 0). These thermodynamic profiles provide critical guidance for selecting optimal surface configurations at specific electrode potentials to guide the kinetic studies of the HER (vide infra).

### Interfacial proton-coupled electron transfer kinetics on O-terminated Ti_2_C MXenes

The Volmer step is a representative heterogeneous PCET reaction, where a proton (from H_3_O^+^ in acid) couples with an electron from the electrode to form adsorbed hydrogen (H^*^). While the theoretical framework for HER electrocatalysis has long relied on the well-known thermodynamic descriptor ΔG_H_ (with ΔG_H_ ~ 0 predicting optimal HER activity)^7^, many non-precious metal catalysts exhibit significantly lower activity (compared to Pt) despite binding hydrogen with similar strength^37–39^. This discrepancy underscores the critical role of kinetics in governing the HER reaction. Emerging evidence reveals that dynamic electrified interface phenomena, such as potential-dependent water reorientation and surface polarization-induced charge redistribution, significantly modulate PCET kinetics^5,40^, which challenges the purely thermodynamic model. Standard constant-charge DFT calculations typically exhibit a variation of the electrode Fermi energy throughout the redox reaction pathway associated with PCET^41^. Given that the strength and configuration of the electric field in the EDL, induced by the applied potential, can significantly influence both the kinetics and thermodynamics of the reaction, maintaining a constant electrode potential becomes essential for the accurate PCET process investigation^5^.

Here, we performed constant-potential constrained molecular dynamics (CP-CMD) simulations to investigate PCET reactions at the electrified O-terminated Ti_2_C MXene/water interface. The simulations employed a grand-canonical electronic structure framework, which explicitly controls the applied potential by dynamically adjusting the number of electrons via an external potentiostat. This approach allows the work function to be tuned relative to the target potential, thereby rigorously accounting for the potential-dependent reorganization of the electrocatalytic interface, resulting in potential-dependent thermodynamics and kinetics.

**Fig. 3:**
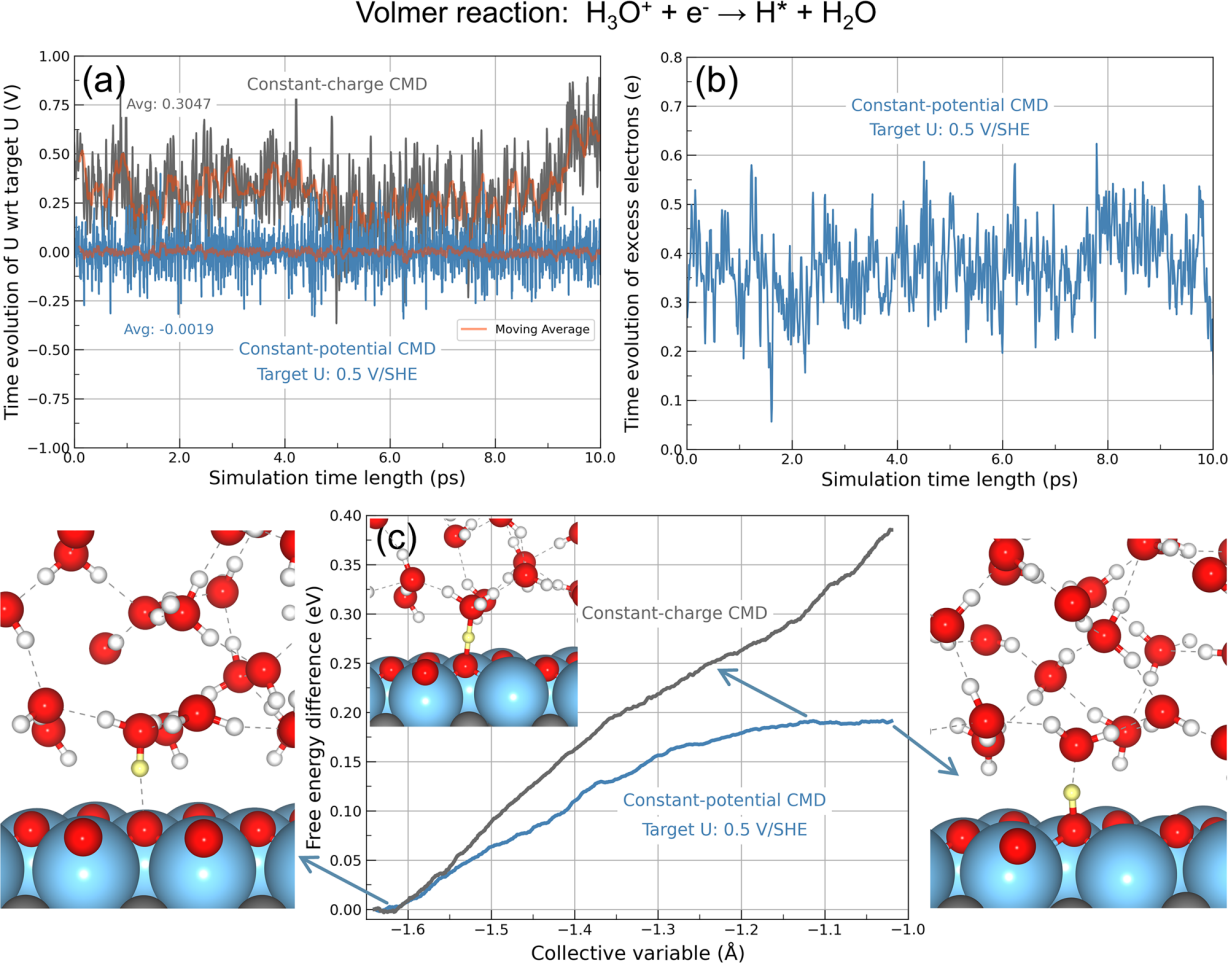
CMD simulations of Volmer step. CMD simulations of Volmer step at the electrified O-terminated Ti_2_C MXene/water interface. **a** The electrode potential evolved during CMD simulations with respect to the target potential 0.5 V/SHE. Blue line: constant-potential CMD simulation; Black line: constant-charge CMD simulation with zero net excess electrons. The moving average (red line) is calculated over a 100-step sliding window. **b** The evolution of the excess electrons for the simulated system to maintain the target potential 0.5 V/SHE during the constant-potential CMD simulation. **c** Free energy profiles for the Volmer step from constant-potential (blue line) and constant-charge (black line) CMD simulations. Key configurations (initial, transition and final states) during constant-potentail CMD simulation are highlighted with corresponding atomic structures. The atomic hydrogen participating in the PCET process is color-coded in yellow for better visual identification.

We started with the Volmer step as shown in Fig. 3. The target potential was set to 0.5 V/SHE for the O-terminated Ti_2_C MXene system, based on the thermodynamically stable phases under pH = 0 condition shown in Fig. 2b, c. The time-resolved electrode potential for the electrified interface that evolved during the CP-CMD simulation is shown by the blue line in Fig. 3a. The electrode potential regulated by the computational potentiostat oscillates around the target potential of 0.5 V/SHE with a mean deviation approaching zero (−0.0019 V), demonstrating the robust control capability of our CP-CMD simulation methodology. The computational potentiostat maintains the constant electrode potential by dynamically adjusting the system’s excess electrons, as shown in Fig. 3b. The excess electrons fluctuate between 0.1 and 0.6 e, demonstrating the effective implementation of the grand-canonical ensemble approach in our simulations. For comparative analysis, we additionally performed constant-charge simulations with zero net excess electrons for the interface, with the corresponding time-resolved electrode potential profile depicted by the black line. The electrode potential during the Volmer reaction process exhibits significant variations, deviating from the target potential by approximately −0.25 to 0.8 V/SHE, which highlights the inherent limitations of constant-charge CMD simulations in maintaining potential control during a PCET event. Free energy profiles for the Volmer step, derived by integrating the mean constraint force along the reaction coordinate, are compared for constant-potential and constant-charge CMD simulations shown in Fig. 3c. While both CMD simulations identify the Volmer step as an endergonic process at the O-terminated Ti_2_C MXene/water interface, the constant-charge approach yields higher free energy difference (~0.4 vs ~0.2 eV). The transition state for the PCET process obtained from the CP-CMD simulation is shown in the inset. The endergonic Volmer step at the electrified O-terminated Ti_2_C MXene/water interface suggests that proton solvation in the outer Helmholtz plane is energetically favored over interfacial PCET to the electrode surface. We calculated the charge density difference (Supplementary Fig. 3) between the initial structure for the Volmer step on the O-terminated Ti_2_C MXene/water interface with and without an external electric field. The charge density difference reveals that the excess electrons are delocalized over both the surface oxygen atoms and the interfacial water network. The delocalized electron density enhances the overall electrostatic environment of the interfacial region and strengthens the hydrogen bonding network. This electron spillover into the water network partially screens the positive charge of the approaching hydronium ion and stabilizes negatively charged oxygen sites, thereby reducing the electrostatic repulsion and reorganization energy required for proton transfer. Consequently, the activation barrier for the Volmer step is lowered. The constant-potential simulation reveals how interfacial electric fields from controlled excess electrons stabilize adsorbed hydrogen and lower activation energies, demonstrating the necessity of potential-controlled methods for modeling electrocatalytic PCET processes.

**Fig. 4:**
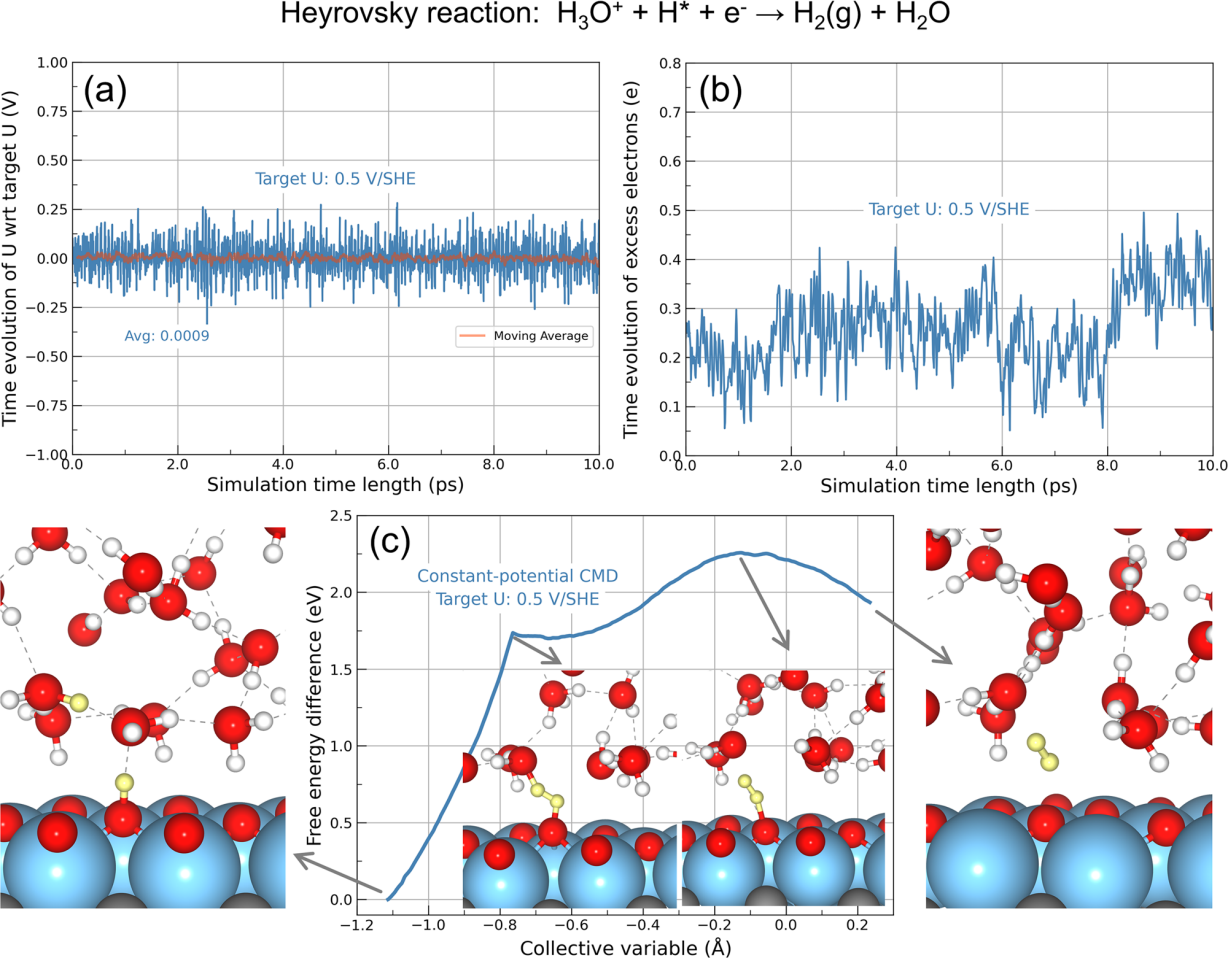
CMD simulations of Heyrovsky step. Constant-potential CMD simulations of Heyrovsky step at the electrified O-terminated Ti_2_C MXene/water interface. **a** The electrode potential evolved during constant-potential CMD simulations with respect to the target potential 0.5 V/SHE. The moving average (red line) is calculated over a 100-step sliding window. **b** The evolution of the excess electrons for the simulated system to maintain the target potential 0.5 V/SHE during the constant-potential CMD simulation. **c** Free energy profiles for the Heyrovsky step from constant-potential CMD simulations. Key configurations (initial, transition and final states) during constant-potentail CMD simulation are highlighted with corresponding atomic structures. The atomic hydrogen participating in the PCET process is color-coded in yellow for better visual identification.

The low hydrogen coverage and the wide inter-oxygen spacing on the O-terminated Ti_2_C MXene surface favor an Eley-Rideal mechanism for hydrogen evolution, where adsorbed hydrogen atoms (H^*^) directly react with solvated protons (H_3_O^+^) from the electrolyte, (i.e., the Heyrovsky step)^42–44^ Therefore, we investigated the Heyrovsky step (as shown in Fig. 4) at the electrified O-terminated Ti_2_C MXene/water interface, which follows the Volmer step. The time-resolved electrode potential (Fig. 4a) during the CP-CMD simulation fluctuates around the target potential of 0.5 V/SHE with a time-averaged value close to zero, demonstrating effective electrode potential control. The system’s excess electrons during the simulation (Fig. 4b) vary from ~0.1 to ~0.5 e.

The free energy profiles obtained from the CP-CMD simulations reveal that the Heyrovsky reaction at the electrified O-terminated Ti_2_C MXene/water interface proceeds via two distinct transition states (Fig. 4c). The first transition state, corresponding to the PCET process, exhibits an activation energy of 1.7 eV, whereas the subsequent transition state, associated with the desorption of molecular hydrogen, has an activation energy of 0.5 eV. The high activation energy originates from the concerted rearrangement of hydrogen bond networks at the MXene/water interface coupled with O-H bond cleavage in the hydronium ion. To understand why H_2_ desorption has a lower barrier despite following the high-energy PCET step, we calculated the radial distribution functions (RDFs) between the reacting species and interfacial water molecules at the two transition states (Supplementary Fig. 4). The RDF analysis reveals that the charged/polar species (H_3_O^+^ + H^*^) at the first transition state exhibit stronger solvation, requiring extensive desolvation and hydrogen bond reorganization. In contrast, the neutral H_2_ molecule at the second transition state shows a flattened RDF distribution, indicating weak interactions with interfacial water that allow facile desorption with minimal reorganization energy. The energetic cost is thus front-loaded into the PCET step, while H_2_ release benefits from reduced water-solute interactions. The reaction is strongly endergonic with an overall free energy increase of ~2.0 eV. These results demonstrate that the Heyrovsky step exhibits sluggish kinetics, confirming it as the rate-determining step for the HER on O-terminated Ti_2_C MXene under acidic conditions, consistent with experimental observations^45,46^.

### Interfacial proton-coupled electron transfer kinetics on mixed O-/OH-terminated Ti_2_C MXenes

**Fig. 5:**
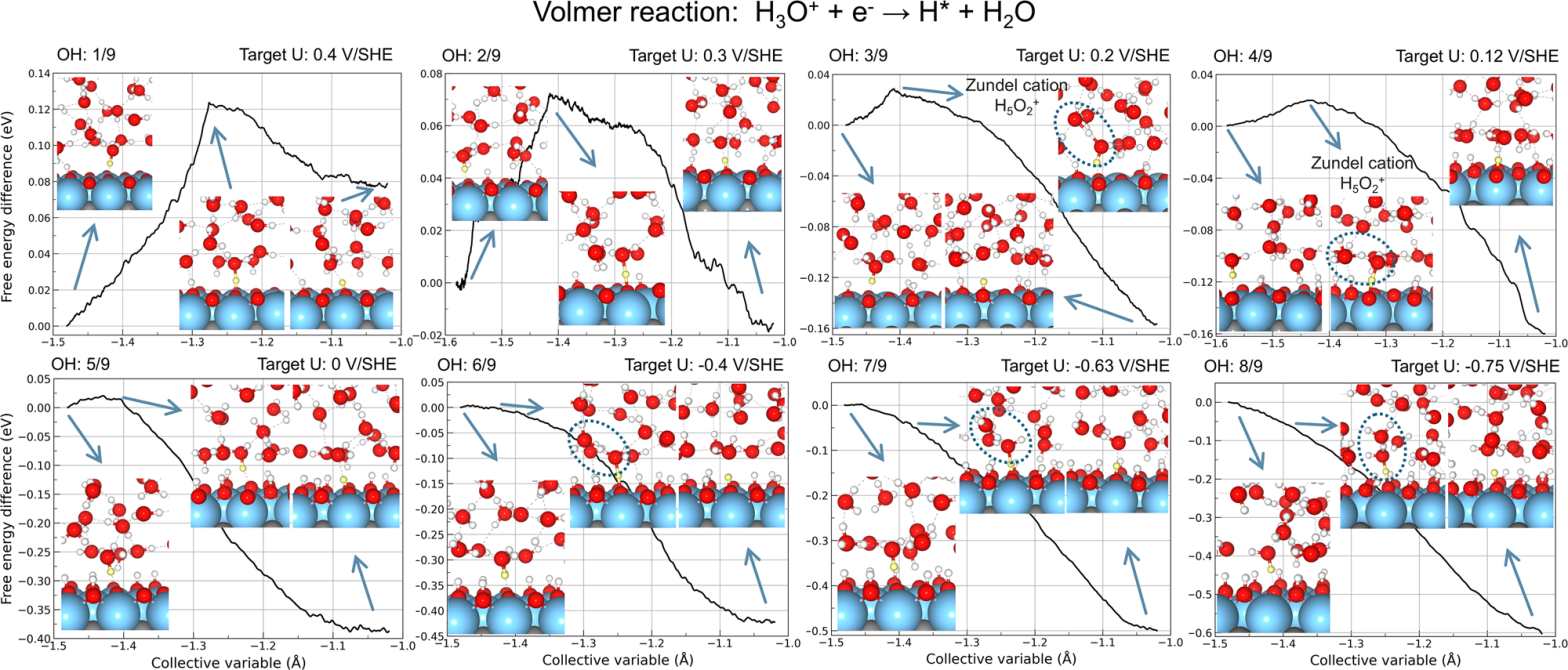
Free energy profiles for the Volmer step on each mixed O-/OH-terminated Ti_2_C MXenes. Key configurations (initial, transition and final states) during constant-potentail CMD simulation are highlighted with corresponding atomic structures. The atomic hydrogen participating in the PCET process is color-coded in yellow for better visual identification.

The Volmer step at the electrified mixed O-/OH-terminated Ti_2_C MXene/water interfaces was systematically investigated for varying OH coverage using constant-potential CMD simulations. For each coverage, the applied electrode potential was determined based on the thermodynamically stable phases under acidic conditions (pH = 0), as identified in the surface Pourbaix diagram (Fig. 2b, c). The free energy profiles for the Volmer step on each mixed O-/OH- terminated Ti_2_C MXenes are shown in Fig. 5. The time evolution of the electrode potential with respect to each target potential and the number of the system’s excess electrons evolved during each CP-CMD simulation are shown in Supplementary Fig. 5. At an OH coverage of 1/9 with an applied electrode potential of 0.4 V/SHE, the free energy profile shows that the Volmer step is slightly endergonic with a low barrier of ~0.12 eV, which exhibits slightly improved kinetics compared to the pure O-terminated Ti_2_C MXene (see Fig. 3c). The transition state structure (see the inset figure) reveals a concerted PCET process, characterized by the simultaneous proton migration from the outer Helmholtz plane and the donation of an electron from the electrode. With increasing OH coverage in steps of 1/9 up to 8/9, the Volmer step becomes exergonic. The exergonic character intensifies with higher OH coverage, reaching a substantial free energy change of −0.6 eV at 8/9 OH coverage under an applied electrode potential of −0.75 V/SHE. However, this trend is not due to an inherent enhancement in the hydrogen adsorption capability of the MXene surface with higher OH termination. Rather, the exergonic character is primarily driven by the negative applied electrode potential, which thermodynamically favors proton discharge. The free energy profiles for the intermediate coverages (2/9 to 5/9 OH) demonstrate nearly barrierless kinetics, with very low activation energies. At the OH coverages ranging from 6/9 to 8/9, the Volmer step becomes thermodynamically spontaneous and kinetically barrierless, indicating a fully facile PCET process for the Volmer step.

By visual analysis of key intermediate structures, we found that the transition states for OH coverage of 1/9 to 5/9 are characterized by significant rearrangements of the hydrogen bond network, including the formation of the Zundel cation (${{{{\rm{H}}}}}_{5}{{{{\rm{O}}}}}_{2}^{+}$; see the inset). For the barrierless regime with OH coverage from 6/9 to 8/9, these Zundel cation intermediates are also identified. These observations demonstrate that the reaction mechanism is dominated by dynamic hydrogen bond restructuring mediated by Zundel cation formation. Zundel-type hydrated protons (${{{{\rm{H}}}}}_{5}{{{{\rm{O}}}}}_{2}^{+}$) within the H-bond water networks have been experimentally characterized by advanced atomic force microscopy techniques^47^. Their studies demonstrate that Zundel cations actively participate in interfacial proton transfer, suggesting their potential role as key intermediates in electrocatalytic hydrogen evolution. Our CP-CMD simulations corroborate these experimental observations, revealing a consistent mechanistic pathway involving Zundel cation-mediated proton transfer at the MXene/water interface^47,48^. Moreover, the downhill free energy profiles at the electrified mixed O-/OH-terminated Ti_2_C MXene/water interfaces indicate the facile rearrangements of these proton-sharing water networks at these surfaces.

**Fig. 6:**
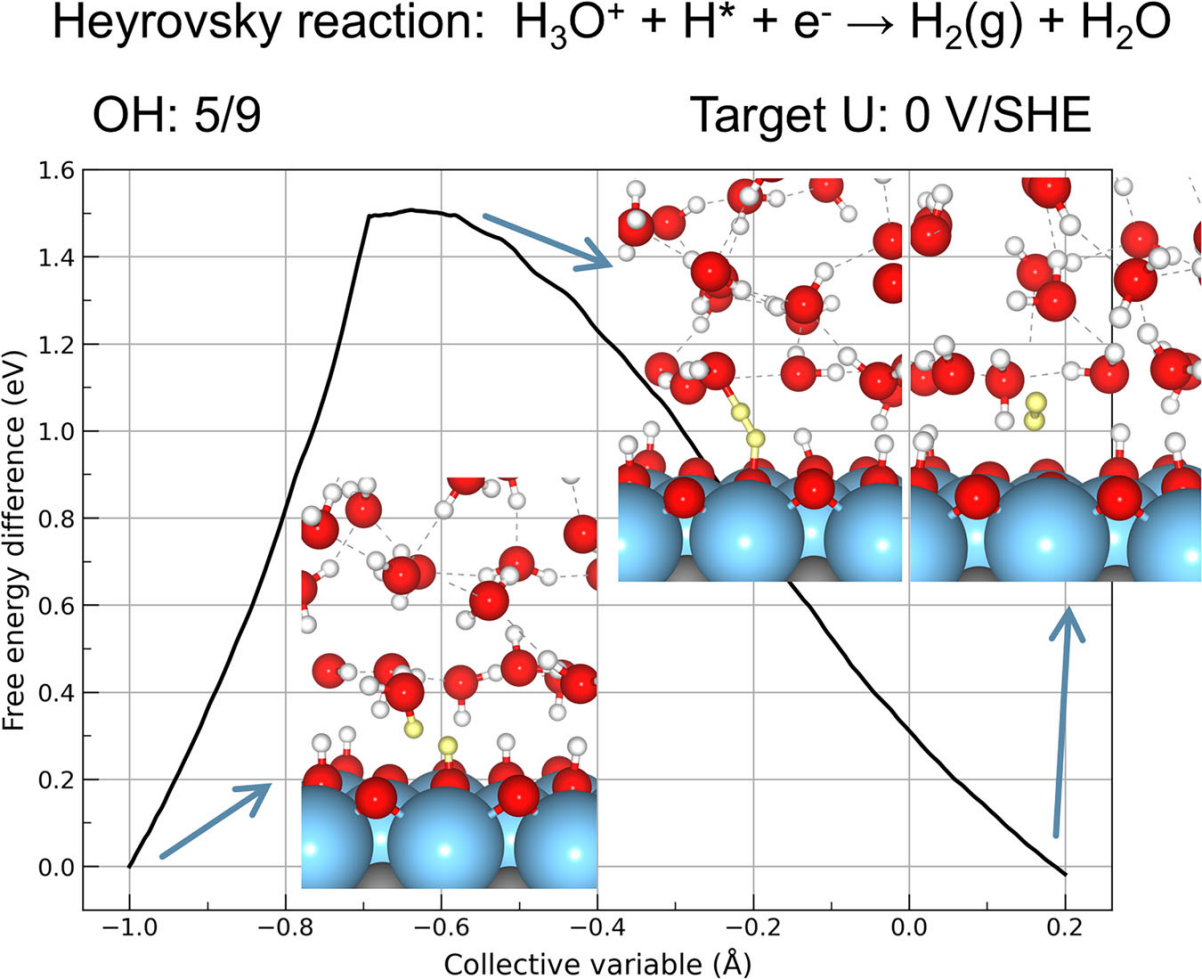
Free energy profile for the Heyrovsky step on mixed O-/OH- terminated Ti_2_C MXenes with OH coverage of 5/9. Key configurations (initial, transition and final states) during constant-potentail CMD simulation are highlighted with corresponding atomic structures. The atomic hydrogen participating in the PCET process is color-coded in yellow for better visual identification.

The Heyrovsky step at the electrified mixed O-/OH-terminated Ti_2_C MXene/water interfaces was investigated on 5/9 OH coverage using constant-potential CMD simulations. As shown in Fig. 6, the reaction is slightly exergonic, indicating a better thermodynamic driving force compared to pure O-terminated Ti_2_C MXene (Fig. 4c). The reaction exhibits a single activation barrier of approximately 1.5 eV, lower than the barrier observed on pure O-terminated Ti_2_C MXene. Analysis of the transition state structure reveals a PCET mechanism, analogous to that identified in the first transition state on the pure O-terminated Ti_2_C MXene. Notably, molecular hydrogen desorption proceeds barrierless, contributing to the overall exergonic character of the Heyrovsky step on mixed O-/OH- terminated Ti_2_C MXene. To complete the coverage series, we performed CP-CMD simulations of the Heyrovsky step on Ti_2_C MXene surfaces with higher OH coverages of 8/9 and full OH termination. The computed free energy profiles (Supplementary Fig. 6) exhibit a similar trend to the profile on mixed O-/OH-terminated Ti_2_C MXene at an OH coverage of 5/9. The results show a monotonic decrease in the Heyrovsky barrier with increasing OH coverage, with the fully OH-terminated surface providing the most favorable reaction pathway under the investigated conditions. This coverage-dependent behavior highlights the critical role of surface hydroxylation in modulating the catalytic activity of Ti_2_C MXene for hydrogen evolution.

### Improved interfacial H-bond networks by terminating OH species

**Fig. 7:**
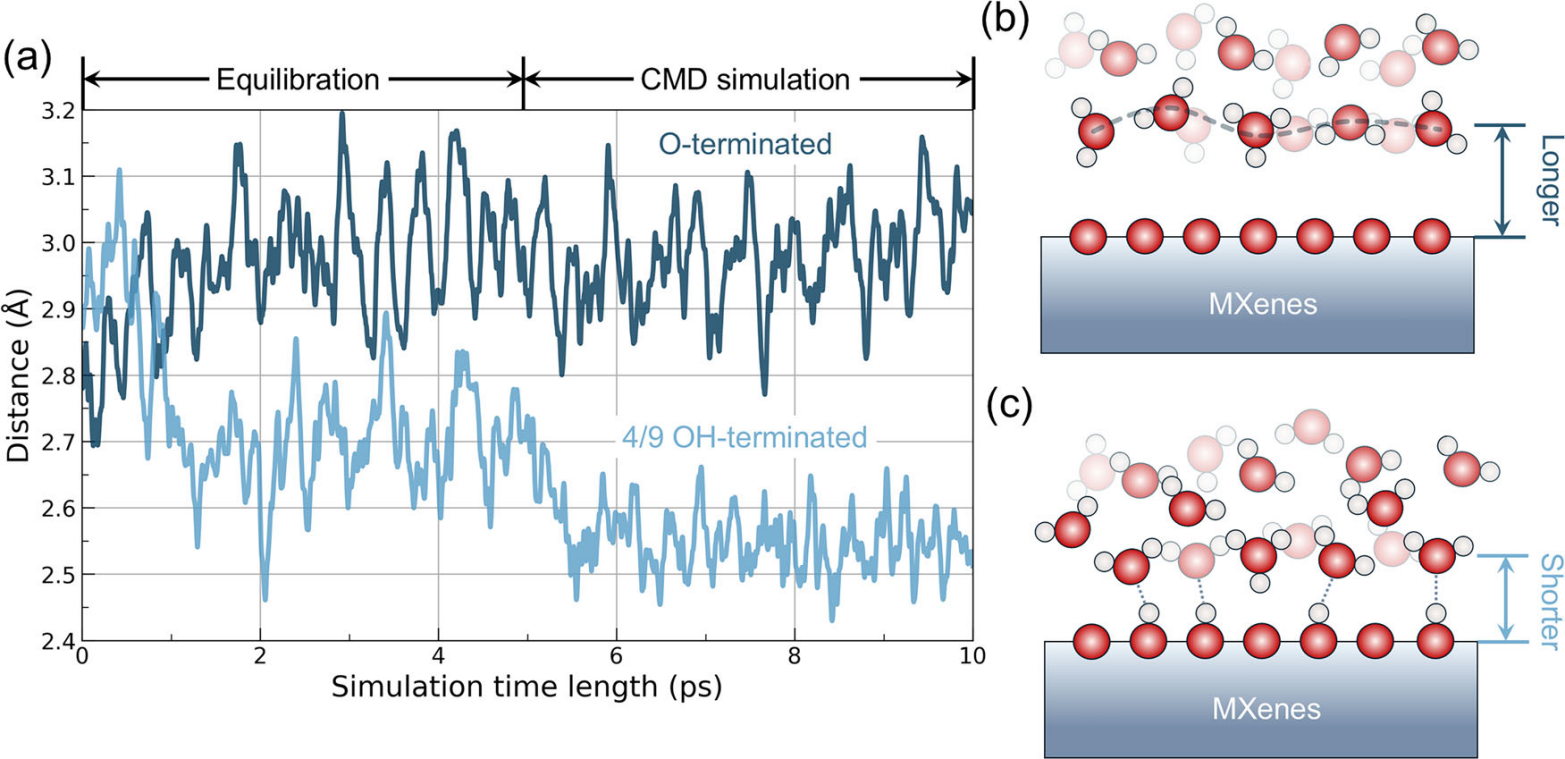
Time-evolved interfacial distances. **a** Time evolution of the distance between the interfacial bottom water layer and the surface termination oxygen species on O-terminated Ti_2_C MXenes (dark blue) and mixed O-/OH-terminated Ti_2_C MXenes with 4/9 OH coverage (light blue) during constant-potential CMD simulations of the Volmer step. The entire constant-potential simulation includes 5 ps equilibration followed by 5 ps CMD simulation. Schematic figure for calculating the distance between the bottom-layer water oxygen and the surface-terminated oxygen on **b** pure O-terminated Ti_2_C MXenes; **c** mixed O-/OH-terminated Ti_2_C MXenes. The hydrogen bonds between the interfacial water molecules and the surface termination OH species on MXenes surface are depicted as dashed line in (**c**).

Recent advances in electrocatalysis have highlighted the critical role of interfacial H-bond networks within the EDL in governing interfacial PCET kinetics^13–15^. To elucidate the improved PCET kinetics on mixed O-/OH-terminated Ti_2_C MXenes, we systematically analyzed the interfacial H-bond networks by tracing the time evolution of the distances between the interfacial bottom water layer and the surface termination oxygen species (as shown in Fig. 7a). Our CP-CMD simulation of the PCET process includes 5 ps of equilibration followed by 5 ps of reaction simulation. Notably, the distances between the basal water layer and the surface oxygen species are significantly shorter on mixed O-/OH-terminated Ti_2_C MXene compared to pure O-terminated surfaces, both during the equilibration process and throughout the PCET process. For clarity, Fig. 7a presents data for the 4/9 OH coverage case, while complete results for all coverages are provided in Supplementary Fig. 7. As shown in Supplementary Fig. 7, the time-evolved interfacial distances are color-coded by coverage, revealing a systematic downward shift with increasing OH coverage despite thermal fluctuations in individual trajectories. This progressive reduction demonstrates that surface OH groups consistently brings the interfacial water layer closer to the MXene surface, enhancing H-bond network connectivity. To further decouple the effects of surface chemistry and applied electrode potential, we performed additional CP-CMD simulations for pure O-terminated Ti_2_C MXene at the target electrode potential of 0 and −0.5 V/SHE, respectively. The time evolution of interfacial distances under the three target *U* (Supplementary Fig. 8) exhibits highly overlapping profiles. The potential-independent behavior strongly suggests that the interfacial water structure is primarily governed by surface chemical compositions.

The pronounced gap observed between water molecules and the pure O-terminated MXene surface at the interface directly results in greatly reduced connectivity of hydrogen bond networks and therefore an increased hydrogen transfer barrier in the interfacial region (see the schematic figure in Fig. 7b). In contrast, the presence of OH terminations reduces this interfacial gap by approximately 0.4 Å (Fig. 7c), thereby significantly enhancing H-bond network connectivity and facilitating proton transfer at the interface. The OH terminations serve as structural anchors that reorganize the interfacial water network within the EDL, transforming discontinuous H-bond arrangements into an interconnected H-bond network. This restructuring reduces the energetic penalty for hydronium ion dehydration and stabilizes key proton-transfer intermediates (e.g., Zundel cations), thereby lowering the activation energy barrier for both the Volmer and Heyrovsky steps compared to pure O-terminated MXene surfaces. To quantify this effect, we analyzed the occurrence frequency of Zundel cations along the CP-CMD trajectories as a function of OH coverage (Supplementary Fig. 9a). The Zundel cation is identified using geometric criteria based on O-O and O-H distances (see Supplementary Fig. 9 for details). The results reveal a non-monotonic dependence on OH coverage, with the maximum Zundel population occurring at intermediate coverage (3/9). At higher OH coverages, Zundel occurrences decrease due to the earlier onset of PCET along the reaction coordinate, manifesting as a more facile, nearly barrierless hydrogen adsorption process. Furthermore, cross-correlation analysis between Zundel presence and the collective variable for the Volmer step (Supplementary Fig. 9b) demonstrates that Zundel formation occurs preferentially near the transition state region, with the peak preceding the barrier. This temporal correlation provides direct evidence that Zundel cations facilitate transition state crossing by pre-organizing the proton-transfer pathway and reducing the reorganization energy required for proton transfer from hydronium to the surface.

## Discussion

Our results demonstrate that mixed O/OH-terminated surfaces are thermodynamically stable under the reducing conditions (negative electrode potentials vs SHE) relevant for the HER. This finding not only explains the active surface structure governing the reaction kinetics but also has crucial implications for the electrochemical stability of the MXene catalyst. The preservation of mixed terminations under operational conditions suggests that the material is in a stable thermodynamic state, thereby mitigating degradation pathways such as dissolution or oxidation. Specifically, the dissolution of Ti atoms, which requires oxidizing conditions, is thermodynamically suppressed at the negative potentials applied during HER. Consequently, the inherent stability of the O/OH-terminated surface under working conditions enhances the durability of the MXene electrocatalyst, addressing a common challenge faced by many non-precious metal-based catalysts.

Computational studies of MXene HER have focused on optimizing hydrogen binding thermodynamics (ΔG_H_) using static DFT calculations^49–51^, which cannot capture interfacial dynamics, realistic potential control, or kinetic barriers. Our CP-CMD simulations investigate HER mechanisms on Ti_2_C MXenes under grand canonical ensemble conditions, explicitly modeling the electrified interface and PCET processes. This approach captures dynamic phenomena inaccessible to static calculations while maintaining realistic electrochemical conditions through dynamic charge adjustment–critical for PCET processes where charge transfer and potential are intrinsically coupled.

The reduced interfacial separation with mixed O/OH termination arises from complementary hydrogen bonding functionalities: surface O atoms act as H-bond acceptors while surface OH groups act as H-bond donors, creating a diverse binding environment that enables better organization of the interfacial water layer compared to pure O-termination. Analysis of coverage-dependent trends (Supplementary Fig. 7) shows that intermediate OH coverages (around 4/9 to 6/9) provide optimal balance between these complementary functionalities, maximizing interfacial water ordering and H-bond network connectivity. The critical role of OH termination species in improving the PCET kinetics aligns with prior studies of adsorbed hydroxyl species on Ru sites in Pt-Ru alloys, where OH_*ad*_ species similarly enhances H-bond connectivity and boosts catalytic activity for hydrogen electrocatalysis compared to pure Pt^14^. These findings establish a direct structure-activity relationship between H-bond network connectivity and PCET kinetics - a correlation that extends beyond the traditional thermodynamic descriptor ΔG_H_. While our study focuses on acidic HER, the mechanistic principle of interfacial optimization through surface functionalization may extend to other pH regimes. Recent work on MXene quantum dots for alkaline HER demonstrates that the surface functionalization enhances H_2_O adsorption at the electrode interface, thereby facilitating H_2_O dissociation^52^. This observation parallels our finding that surface OH groups optimize the interfacial water structure in acidic media by enhancing H-bond connectivity and stabilizing proton-transfer intermediates. These results suggest that appropriate surface functionalization can improve HER activity across different pH ranges by adapting to the dominant reaction pathway at each condition. Collectively, these findings provide fundamental insights into how EDL restructuring governs electrocatalytic kinetics, thereby introducing a crucial and often overlooked factor for understanding rate limitations in PCET process involved in HER, hydrogen oxidation reaction, CO_2_ and N_2_ electroreduction. Meanwhile, our developed constant-potential CMD protocol establishes a rigorous computational framework for exploring electrochemical interfaces at the atomic scale.

## Conclusions

Through constant-potential constrained molecular dynamics (CP-CMD) simulations, we demonstrate that mixed O/OH-terminated Ti_2_CT_2_ MXenes significantly enhance HER kinetics compared to pure O-terminated surfaces by restructuring the EDL into continuous H-bond networks. The OH groups are not mere spectators but serve as structural anchors, shortening the water-MXene interface distance and stabilizing key proton-transfer intermediates like Zundel cations, thereby reducing activation barriers for both the Volmer and Heyrovsky steps. While high OH coverages require negative electrode potentials for thermodynamic stability (e.g., −0.75 V/SHE for 8/9 coverage at pH 0), moderate coverages (5/9 and 6/9) that are stable near typical HER operating conditions already provide substantial kinetic benefits with nearly barrierless Volmer steps. This work establishes interfacial H-bond connectivity as a critical kinetic regulator of PCET processes. Our findings not only provide a new design principle for MXene-based electrocatalysts through the engineering of functional terminations, but also demonstrate how dynamic interfacial processes can be rigorously captured by CP-CMD simulations, enabling precise modeling of electrified interfaces under operational electrocatalytic conditions.

## Methods

### Computational models

To systematically investigate the stability of Ti_2_CT_2_ (T=O, OH) MXenes with varying hydroxyl coverage, we employed a *p*(3 × 3) supercell containing nine unit cells of Ti_2_CT_2_, which enables systematic variation of OH coverage from 0 to 1 in increments of 1/9. For mixed-termination systems (containing both O and OH groups), we ensured mirror symmetry between the top and bottom surface terminations to avoid artificial surface dipoles. For each mixed-termination MXene, all possible configurations of O and OH with different distributions have been explored to identify the most favorable arrangements.

### Electronic structure calculations

DFT calculations were performed with the Vienna Ab-initio Simulation Package (VASP)^53^. The Kohn-Sham orbitals were expanded with plane waves using a 450 eV energy cut-off and the interaction between the valence electrons and the cores was described with the plane augmented wave (PAW) approach^54,55^. The number of valence electrons considered in the calculations are 1 (H), 4 (C), 6 (O) and 4 (Ti). The exchange-correlation effects were described within the generalized gradient approximation according to Perdew, Burke and Ernzerhof^56^. The van der Waals interactions were accounted for using the Grimme’s DFT-D3 approach^57^. A 4 × 4 × 1 *Γ*-centered k-mesh was employed for geometry optimization, while *Γ*-point sampling was used for ab initio molecular dynamics (AIMD) simulations. The electronic self-consistency loop stops when the total energy change between two subsequent steps is smaller than 1 × 10^−5^ eV. Structures were optimized with the conjugate gradient method and geometries were considered to be converged when the largest force on the atoms in the calculated system is smaller than 0.02 eV Å^−1^.

### Electrified interface modeling with hybrid solvation

The electrified solid-liquid interface was modeled using a hybrid approach that combines explicit water molecules with an implicit solvent representation as implemented in VASPsol^++^^58^. Specifically, approximately four layers of explicit water molecules (totaling 30 H_2_O molecules) were placed above the MXene surface to capture the hydrogen-bonding network, water orientation and interfacial polarization. The thickness of the explicit water film was roughly 12 Å to ensure the bulk liquid water region^59^. This explicit solvent layer was coupled with an implicit solvent model to account for bulk electrolyte effects and enable efficient constant-potential simulations. To maintain numerically reliable electrostatic potential decay at the cell boundaries^58^, the simulation cell was constructed with a 30 Å vacuum separation between periodic structures of water solvated MXene. To simulate the acidic environment, one hydronium ion (H_3_O) was included in the water film. Hydrogen bonds were defined using the following geometric criteria: O⋯O distance ≤3.5 Å, 1.2 Å≤ H⋯O distance ≤2.1 Å, and O–H⋯O angle ≥135^∘^.

### Grand-canonical modeling of electrified interface stability

The stable surface terminations of the MXenes under electrochemical conditions were determined through a grand-canonical approach, accounting for both electrode potential and pH-dependent thermodynamics of the electrode/electrolyte interface^35^. Thermodynamic equilibrium was maintained across three coupled subsystems, e.g., bulk electrode, bulk electrolyte and the electrified interface region. The thermodynamically stable surface under given electrochemical conditions was determined by the lowest free energy of adsorption per surface area, which is calculated as:

1 $$\Delta \gamma =\frac{1}{{{{\rm{A}}}}_{{{{\rm{s}}}}}}({{{\rm{{G}}}}}_{{{{\rm{mOnOH/MXene}}}}}^{{{\rm{{interf}}}}}-{{{\rm{{G}}}}}_{({{{\rm{m}}}}+{{{\rm{n}}}}){{{\rm{O/MXene}}}}}^{{{\rm{{interf}}}}}-{{{\rm{n}}}}{\widetilde{\mu }}_{{{{\rm{H}}}}})$$

where ${{{{\rm{G}}}}}_{{{{\rm{mOnOH/MXene}}}}}^{{{{\rm{interf}}}}}$ and ${{{{\rm{G}}}}}_{({{{\rm{m}}}}+{{{\rm{n}}}}){{{\rm{O}}}}/{{{\rm{MXene}}}}}^{{{{\rm{interf}}}}}$ are the free energy of the interface region for Ti_2_CT_2_ (T = O, OH) MXenes with m O and n OH termination groups and for the MXene with fully O terminated configuration, respectively, and ${{{\rm{n}}}}{\widetilde{\mu }}_{{{{\rm{H}}}}}$ is the electrochemical potential of solvated hydrogen. Given by the equilibrium of the reaction

2 $${{{{\rm{H}}}}}^{+}+{{{{\rm{e}}}}}^{-}\rightleftarrows \frac{1}{2}{{{{\rm{H}}}}}_{2}$$

at all pH values and temperatures with H_2_ at 1 bar pressure, the electrochemical potential of the hydrogen can be written as^35^:

3 $${\widetilde{\mu }}_{{{{\rm{H}}}}}={\widetilde{\mu }}_{{{{{\rm{H}}}}}^{+}({{{\rm{aq}}}})}+{\widetilde{\mu }}_{{{{{\rm{e}}}}}^{-}}=\frac{1}{2}{\mu }_{{{{{\rm{H}}}}}_{2}({{{\rm{g}}}})}-{{{{\rm{eU}}}}}({{{\rm{V/SHE}}}})-{{{{k}}}}_{{{{\rm{B}}}}}{{{\rm{Tln}}}}(10){{{\rm{pH}}}}$$

which is the basis of the concept for the CHE proposed by Nørskov et al.^30^ Here, ${\mu }_{{{{{\rm{H}}}}}_{2}({{{\rm{g}}}})}={{{{\rm{E}}}}}_{{{{{\rm{H}}}}}_{2}}^{{{{\rm{tot}}}}}+\Delta {\mu }_{{{{{\rm{H}}}}}_{2}}({{{\rm{T}}}},1\,{{{\rm{bar}}}})$ and the *U*(V/SHE) is the electrode potential given on the standard hydrogen electrode (SHE) scale.

To account for the influence of the electric potential on the energetics of the electrified interface, we systematically varied the excess charge in the simulation cell while allowing the implicit electrolyte (described by the linearized Poisson-Boltzmann equation as implemented in VASPsol^++^^58,60^) to self-consistently adjust counterion concentrations for charge neutrality. The electrode potential (*U*(V/SHE)) was subsequently calculated according to the double reference method^61^, written as

4 $$U({{{\rm{V}}}}/{{{\rm{SHE}}}})=-4.57-{\phi }_{{{{\rm{q}}}}}({{{\rm{f}}}})/{{{\rm{eV}}}}$$

where 4.57 is the theoretical electrode potential of the SHE^58,60^ and −*ϕ*_q_(f)/eV is the work function of the charged system in implicit solvent. To model the acidic condition, the bulk H^+^ concentration was set to 1 mol/L in VASPsol^++^. The (electronic) grand-canonical energy (*Ω*) of the system is therefore corrected for the different number of electrons present in each system and the interaction with the electrolyte, as shown:

5 $$\Omega ={{{{\rm{E}}}}}_{{{{\rm{DFT}}}}}-\Delta {{{n}}}{\mu }_{e}-\Delta {n}{\phi }_{{{{\rm{sol}}}}}$$

where *Δn* is the excess charge of the system, *μ*_*e*_ is the chemical potential of an electron (equal to *ϕ*_*q*_(*f*)) and *ϕ*_*sol*_ is the electrostatic potential of the bulk electrolyte. For each system, the net charge per simulation cell was varied from −0.5 e to +0.5 e in steps of 0.1 e. The potential-dependent grand-canonical energies across the 11 charge states were fit to a quadratic functional^60^:

6 $$E(U)=-\frac{1}{2}C{(U-{U}_{0})}^{2}+{E}_{0}$$

where *C* is the capacitance of the electrified interface, *U*_0_ is the PZC and *E*_0_ is the energy at the PZC. The parabolic relationship captures the mean-field electrostatic response of the double layer, with the curvature yielding the capacitance (*C* = ∂^2^*E*/∂*U*^2^). The fitted quadratic energy-potential relationship for each MXene system was subsequently incorporated into the hydrogen adsorption free energy calculation (Eq. (1)).

### Constant-potential constrained molecular dynamics simulations

Born-Oppenheimer AIMD were performed in the NVT ensemble as implemented using the VASP package. The system temperature was maintained at 298.15 K employing a Nosé-Hoover thermostat^62,63^. To facilitate the integration of the equations of motion, the hydrogen mass was artificially increased to the isotopic mass of tritium, allowing the use of a 1 fs time step for stable numerical integration. The free-energy profile along a defined reaction path was investigated using slow-growth approach^64^ implemented via ab initio constrained molecular dynamics (CMD) simulations in VASP. This method involved gradually varying specified collective variables to probe the system’s response along the reaction path. To compute the free energy difference associated with the reaction event, we employed thermodynamic integration of the free energy gradients derived from the constrained simulations. This approach offers valuable insights into the thermodynamics and kinetics of the studied reaction. Assuming that the reaction path can be described via a set of coordinates *ξ* = {*ξ*_*k*_; *k* = 1,…,*r*}, the free-energy difference between the initial and final states is calculated according to:

7 $$\Delta {A}_{{{{\rm{I\to F}}}}}=\int_{\xi ({{{\rm{I}}}})}^{\xi ({{{\rm{F}}}})}{\left(\frac{\partial A}{\partial \xi }\right)}_{{\xi }^{* }}d\xi$$

where ${\left(\frac{\partial A}{\partial \xi }\right)}_{{\xi }^{* }}$ denotes the free-energy gradient evaluated at a particular point along the reaction path, denoted as ξ^*^. The free-energy gradients ${\left(\frac{\partial A}{\partial \xi }\right)}_{{\xi }^{* }}$ were derived via blue-moon ensemble method^65^, which allows for enhanced sampling of configurational space associated with the defined reaction coordinates (or collective variables)^66^. The simulation protocol consisted of 10 ps classical MD pre-equilibration of water layers (TIP3P force field), 5 ps CP-AIMD equilibration in VASP, followed by 5 ps CP-CMD production runs for free energy calculations. Further details regarding the CMD simulations can be found in Supplementary Information under the Supplementary Methods section of “CP-CMD simulations”.

**Fig. 8:**
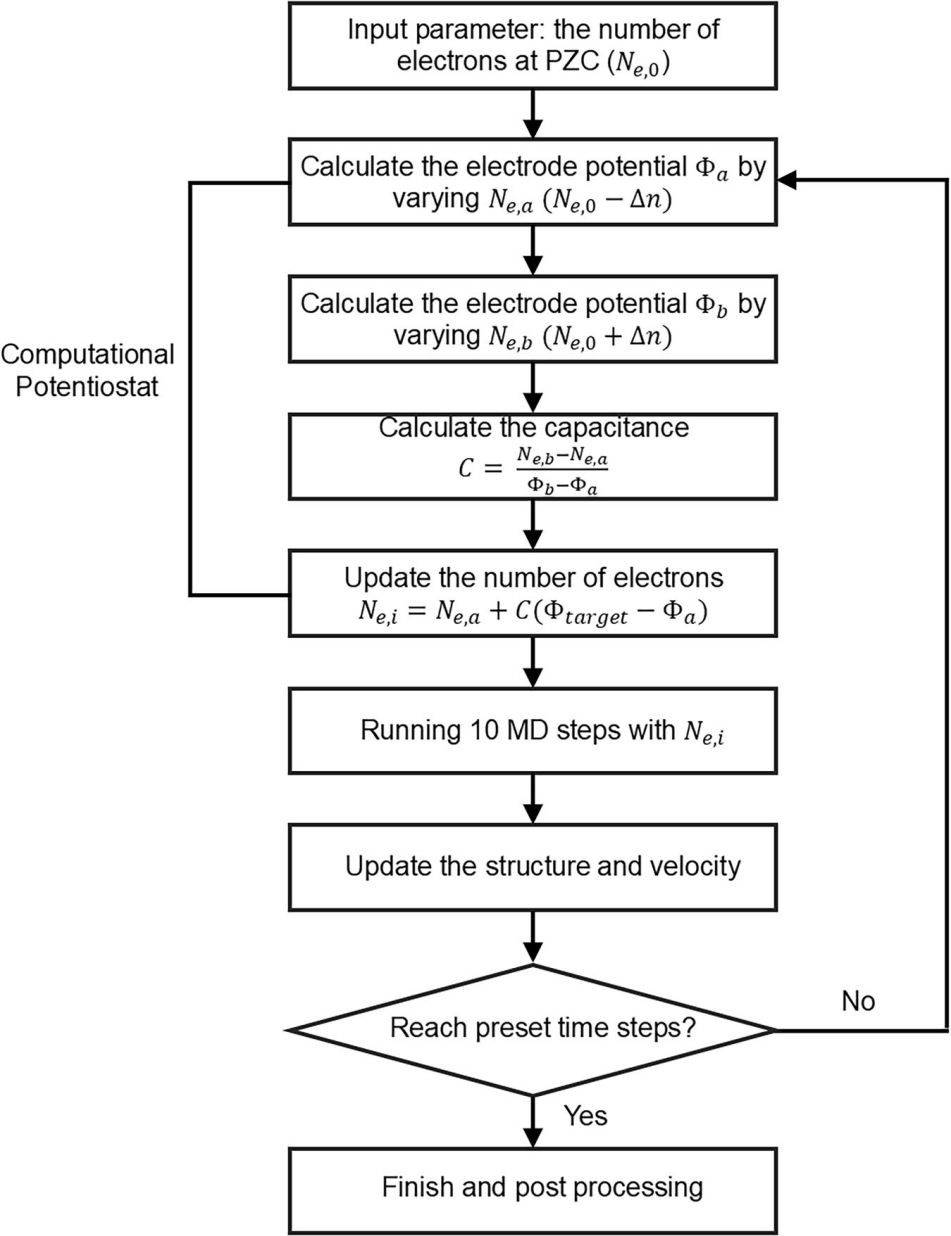
CP-CMD flowchart. Flowchart for performing constant-potential constrained molecular dynamics (CP-CMD) simulations.

To maintain the target potential *Φ*_*target*_ for the electrified interface system during the CMD simulations, we employed a grand-canonical electronic structure approach for explicit control of the applied potential by varying the number of electrons of the electrocatalytic systems. The variation is monitored and controlled by an external potentiostat, resulting in constant-potential constrained molecular dynamics (CP-CMD) simulations. Consequently, the electrode potential *Φ* evolved during CMD simulations flucturates around *Φ*_*target*_, and 〈*Φ*〉 = *Φ*_*target*_, as required for any potentiostat scheme^67^. Figure 8 illustrates the procedural flowchart for performing CP-CMD simulations. While the depicted approach is specifically used for CP-CMD in this study, it is also applicable to general molecular dynamics (MD) simulations.

## Supplementary information

Transparent Peer Review file

Supplementary Information

## Data Availability

The data underlying the findings of this study are available within the article and its Supplementary Data. All the calculated structure, trajectory files and source data generated in this study have been deposited in Zenodo (10.5281/zenodo.17132591).

## References

[1] Chu, S. & Majumdar, A. Opportunities and challenges for a sustainable energy future. *Nature* **488**, 294–303 (2012). 22895334 10.1038/nature11475

[2] Gasteiger, H. A. & Marković, N. M. Just a dream-or future reality? *Science* **324**, 48–49 (2009). 19342578 10.1126/science.1172083

[3] Costentin, C. Electrochemical approach to the mechanistic study of proton-coupled electron transfer. *Chem. Rev.* **108**, 2145–2179 (2008). 18620365 10.1021/cr068065t

[4] Hammes-Schiffer, S. Proton-coupled electron transfer: classification scheme and guide to theoretical methods. *Energy Environ. Sci.* **5**, 7696–7703 (2012).

[5] Warburton, R. E., Soudackov, A. V. & Hammes-Schiffer, S. Theoretical modeling of electrochemical proton-coupled electron transfer. *Chem. Rev.* **122**, 10599–10650 (2022). 35230812 10.1021/acs.chemrev.1c00929

[6] Nørskov, J. K. et al. Trends in the exchange current for hydrogen evolution. *J. Electrochem. Soc.* **152**, J23 (2005).

[7] Greeley, J., Jaramillo, T. F., Bonde, J., Chorkendorff, I. & Nørskov, J. K. Computational high-throughput screening of electrocatalytic materials for hydrogen evolution. *Nat. Mater.* **5**, 909–913 (2006). 17041585 10.1038/nmat1752

[8] Sheng, W. et al. Correlating hydrogen oxidation and evolution activity on platinum at different pH with measured hydrogen binding energy. *Nat. Commun.* **6**, 1–6 (2015). 10.1038/ncomms6848 25569511

[9] Zheng, J., Sheng, W., Zhuang, Z., Xu, B. & Yan, Y. Universal dependence of hydrogen oxidation and evolution reaction activity of platinum-group metals on pH and hydrogen binding energy. *Sci. Adv.* **2**, e1501602 (2016). 27034988 10.1126/sciadv.1501602 PMC4803484

[10] Ledezma-Yanez, I. et al. Interfacial water reorganization as a pH-dependent descriptor of the hydrogen evolution rate on platinum electrodes. *Nat. Energy* **2**, 1–7 (2017).

[11] Rebollar, L. et al. "Beyond adsorption” descriptors in hydrogen electrocatalysis. *ACS Catal.* **10**, 14747–14762 (2020).

[12] Berg, N., Bergwinkl, S., Nuernberger, P., Horinek, D. & Gschwind, R. M. Extended hydrogen bond networks for effective proton-coupled electron transfer (PCET) reactions: the unexpected role of thiophenol and its acidic channel in photocatalytic hydroamidations. *J. Am. Chem. Soc.* **143**, 724–735 (2021). 33423466 10.1021/jacs.0c08673

[13] Wang, T. et al. Enhancing oxygen reduction electrocatalysis by tuning interfacial hydrogen bonds. *Nat. Catal.* **4**, 753–762 (2021).

[14] Li, P. et al. Hydrogen bond network connectivity in the electric double layer dominates the kinetic pH effect in hydrogen electrocatalysis on Pt. *Nat. Catal.* **5**, 900–911 (2022).

[15] Sun, Q. et al. Understanding hydrogen electrocatalysis by probing the hydrogen-bond network of water at the electrified Pt–solution interface. *Nat. Energy* **8**, 859–869 (2023).

[16] Magnussen, O. M. & Groß, A. Toward an atomic-scale understanding of electrochemical interface structure and dynamics. *J. Am. Chem. Soc.* **141**, 4777–4790 (2019). 30768905 10.1021/jacs.8b13188

[17] Chen, L. D. et al. Understanding the apparent fractional charge of protons in the aqueous electrochemical double layer. *Nat. Commun.* **9**, 3202 (2018). 30097564 10.1038/s41467-018-05511-y PMC6086897

[18] Naguib, M. et al. Two-dimensional nanocrystals: two-dimensional nanocrystals produced by exfoliation of Ti3AlC2 (adv. mater. 37/2011). *Adv. Mater.* **23**, 4207–4207 (2011). 10.1002/adma.201102306 21861270

[19] VahidMohammadi, A., Rosen, J. & Gogotsi, Y. The world of two-dimensional carbides and nitrides (mxenes). *Science* **372**, eabf1581 (2021). 34112665 10.1126/science.abf1581

[20] Zhou, J., Dahlqvist, M., Bjork, J. & Rosen, J. Atomic scale design of MXenes and their parent materials—from theoretical and experimental perspectives. *Chem. Rev.* **123**, 13291–13322 (2023). 37976459 10.1021/acs.chemrev.3c00241 PMC10722466

[21] Handoko, A. D. et al. Tuning the basal plane functionalization of two-dimensional metal carbides (mxenes) to control hydrogen evolution activity. *ACS Appl. Energy Mater.* **1**, 173–180 (2017).

[22] Björk, J. & Rosen, J. Functionalizing MXenes by tailoring surface terminations in different chemical environments. *Chem. Mater.* **33**, 9108–9118 (2021).

[23] Natu, V. & Barsoum, M. W. Mxene surface terminations: a perspective. *J. Phys. Chem. C.* **127**, 20197–20206 (2023).

[24] Seh, Z. W. et al. Two-dimensional molybdenum carbide (mxene) as an efficient electrocatalyst for hydrogen evolution. *ACS Energy Lett.* **1**, 589–594 (2016).

[25] Gao, G., O’Mullane, A. P. & Du, A. 2D MXenes: a new family of promising catalysts for the hydrogen evolution reaction. *ACS Catal.* **7**, 494–500 (2017).

[26] Naguib, M. et al. Two-dimensional transition metal carbides. *ACS Nano* **6**, 1322–1331 (2012). 22279971 10.1021/nn204153h

[27] Persson, I. et al. On the organization and thermal behavior of functional groups on Ti3C2 MXene surfaces in vacuum. *2D Mater.* **5**, 015002 (2017).

[28] Ibragimova, R., Puska, M. J. & Komsa, H.-P. pH-dependent distribution of functional groups on titanium-based MXenes. *ACS nano* **13**, 9171–9181 (2019). 31393102 10.1021/acsnano.9b03511 PMC6748675

[29] Niu, K., Chi, L., Rosen, J. & Björk, J. C–h activation of light alkanes on MXenes predicted by hydrogen affinity. *Phys. Chem. Chem. Phys.* **22**, 18622–18630 (2020). 32789324 10.1039/d0cp02471f

[30] Nørskov, J. K. et al. Origin of the overpotential for oxygen reduction at a fuel-cell cathode. *J. Phys. Chem. B* **108**, 17886–17892 (2004). 39682080 10.1021/jp047349j

[31] Van den Bossche, M., Skúlason, E., Rose-Petruck, C. & Jónsson, H. Assessment of constant-potential implicit solvation calculations of electrochemical energy barriers for H2 evolution on Pt. *J. Phys. Chem. C.* **123**, 4116–4124 (2019).

[32] Ringe, S., Hormann, N. G., Oberhofer, H. & Reuter, K. Implicit solvation methods for catalysis at electrified interfaces. *Chem. Rev.* **122**, 10777–10820 (2021). 34928131 10.1021/acs.chemrev.1c00675 PMC9227731

[33] Tian, Y. et al. Theoretical insights on potential-dependent oxidation behaviors and antioxidant strategies of MXenes. *Nat. Commun.* **15**, 10099 (2024). 39572580 10.1038/s41467-024-54455-z PMC11582733

[34] Hörmann, N. G., Marzari, N. & Reuter, K. Electrosorption at metal surfaces from first principles. *npj Comput. Mater.* **6**, 136 (2020).

[35] Groß, A. Reversible vs standard hydrogen electrode scale in interfacial electrochemistry from a theoretician’s atomistic point of view. *J. Phys. Chem. C.* **126**, 11439–11446 (2022).

[36] Mello, G. A., Briega-Martos, V., Climent, V. & Feliu, J. M. Bromide adsorption on Pt (111) over a wide range of pH: Cyclic voltammetry and co displacement experiments. *J. Phys. Chem. C.* **122**, 18562–18569 (2018).

[37] Jaramillo, T. F. et al. Identification of active edge sites for electrochemical H2 evolution from MOS2 nanocatalysts. *science* **317**, 100–102 (2007). 17615351 10.1126/science.1141483

[38] Huang, T.-X. et al. Visualizing the structural evolution of individual active sites in MoS2 during electrocatalytic hydrogen evolution reaction. *Nat. Catal.* **7**, 646–654 (2024).

[39] Lindgren, P., Kastlunger, G. & Peterson, A. A. A challenge to the *G* ~ 0 interpretation of hydrogen evolution. *ACS Catal.* **10**, 121–128 (2019).

[40] Patel, D. M. & Kastlunger, G. Non-Nernstian effects in theoretical electrocatalysis. *Chem. Rev.* **125**, 3378–3400 (2025). 10.1021/acs.chemrev.4c00803 40048413

[41] Chan, K. & Nørskov, J. K. Electrochemical barriers made simple. *J. Phys. Chem. Lett.* **6**, 2663–2668 (2015). 26266844 10.1021/acs.jpclett.5b01043

[42] Li, Y. et al. Mos2 nanoparticles grown on graphene: an advanced catalyst for the hydrogen evolution reaction. *J. Am. Chem. Soc.* **133**, 7296–7299 (2011). 21510646 10.1021/ja201269b

[43] Miu, E. V., McKone, J. R. & Mpourmpakis, G. The sensitivity of metal oxide electrocatalysis to bulk hydrogen intercalation: hydrogen evolution on tungsten oxide. *J. Am. Chem. Soc.* **144**, 6420–6433 (2022). 35289172 10.1021/jacs.2c00825

[44] Zheng, X. et al. Tailoring a local acid-like microenvironment for efficient neutral hydrogen evolution. *Nat. Commun.* **14**, 4209 (2023). 37452036 10.1038/s41467-023-39963-8 PMC10349089

[45] Ran, J. et al. Ti3c2 MXene co-catalyst on metal sulfide photo-absorbers for enhanced visible-light photocatalytic hydrogen production. *Nat. Commun.* **8**, 13907 (2017). 28045015 10.1038/ncomms13907 PMC5512649

[46] Yuan, W. et al. Mxene nanofibers as highly active catalysts for hydrogen evolution reaction. *ACS Sustain. Chem. Eng.* **6**, 8976–8982 (2018).

[47] Tian, Y. et al. Visualizing eigen/zundel cations and their interconversion in monolayer water on metal surfaces. *Science* **377**, 315–319 (2022). 35857595 10.1126/science.abo0823

[48] Schröder, M., Gatti, F., Lauvergnat, D., Meyer, H.-D. & Vendrell, O. The coupling of the hydrated proton to its first solvation shell. *Nat. Commun.* **13**, 6170 (2022). 36257946 10.1038/s41467-022-33650-w PMC9579203

[49] Zhang, J. et al. Single platinum atoms immobilized on an MXene as an efficient catalyst for the hydrogen evolution reaction. *Nat. Catal.* **1**, 985–992 (2018).

[50] Kuznetsov, D. A. et al. Single site cobalt substitution in 2d molybdenum carbide (MXene) enhances catalytic activity in the hydrogen evolution reaction. *J. Am. Chem. Soc.* **141**, 17809–17816 (2019). 31540549 10.1021/jacs.9b08897

[51] Chowdhury, C. et al. Predicting the her activity of sacs on mxenes with simple features and interpretable machine learning models. *J. Mater. Chem. A* **14**, 5349–5365 (2026).

[52] Liu, Y. et al. Mxene quantum dots-co (oh) 2 heterojunction stimulated co2+ *δ* sites for boosted alkaline hydrogen evolution. *Acta Mater.* **283**, 120507 (2025).

[53] Kresse, G. & Hafner, J. Ab initio molecular-dynamics simulation of the liquid–metal–amorphous–semiconductor transition in germanium. *Phys. Rev. B* **49**, 14251–14269 (1994). 10.1103/physrevb.49.14251 10010505

[54] Blöchl, P. E. Projector augmented-wave method. *Phys. Rev. B* **50**, 17953–17979 (1994). 10.1103/physrevb.50.17953 9976227

[55] Kresse, G. & Joubert, D. From ultrasoft pseudopotentials to the projector augmented-wave method. *Phys. Rev. B* **59**, 1758–1775 (1999).

[56] Perdew, J. P., Burke, K. & Ernzerhof, M. Generalized gradient approximation made simple. *Phys. Rev. Lett.* **77**, 3865–3868 (1996). 10062328 10.1103/PhysRevLett.77.3865

[57] Grimme, S. Semiempirical gga-type density functional constructed with a long-range dispersion correction. *J. Comput. Chem.* **27**, 1787–1799 (2006). 16955487 10.1002/jcc.20495

[58] Islam, S., Khezeli, F., Ringe, S. & Plaisance, C. An implicit electrolyte model for plane wave density functional theory exhibiting nonlinear response and a nonlocal cavity definition. *J. Chem. Phys.* **159**, 234117 (2023). 10.1063/5.0176308 38112507

[59] Li, P., Huang, J., Hu, Y. & Chen, S. Establishment of the potential of zero charge of metals in aqueous solutions: different faces of water revealed by ab initio molecular dynamics simulations. *J. Phys. Chem. C.* **125**, 3972–3979 (2021).

[60] Mathew, K., Kolluru, V., Mula, S., Steinmann, S. N. & Hennig, R. G. Implicit self-consistent electrolyte model in plane-wave density-functional theory. *J. Chem. Phys.* **151**, 234101 (2019). 10.1063/1.5132354 31864239

[61] Taylor, C. D., Wasileski, S. A., Filhol, J.-S. & Neurock, M. First principles reaction modeling of the electrochemical interface: Consideration and calculation of a tunable surface potential from atomic and electronic structure. *Phys. Rev. B Condens. Matter Mater. Phys.* **73**, 165402 (2006).

[62] Nosé, S. A unified formulation of the constant temperature molecular dynamics methods. *J. Chem. Phys.* **81**, 511–519 (1984).

[63] Hoover, W. G. Canonical dynamics: equilibrium phase-space distributions. *Phys. Rev. A* **31**, 1695 (1985). 10.1103/physreva.31.1695 9895674

[64] Woo, T. K., Margl, P. M., Blöchl, P. E. & Ziegler, T. A combined car-parrinello QM/MM implementation for ab initio molecular dynamics simulations of extended systems: application to transition metal catalysis. *J. Phys. Chem. B* **101**, 7877–7880 (1997).

[65] Fleurat-Lessard, P. & Ziegler, T. Tracing the minimum-energy path on the free-energy surface. *J. Chem. Phys.* **123**, 084101 (2005). 10.1063/1.1948367 16164276

[66] Levell, Z. et al. Emerging atomistic modeling methods for heterogeneous electrocatalysis. *Chem. Rev.* **124**, 8620–8656 (2024). 38990563 10.1021/acs.chemrev.3c00735

[67] Bonnet, N., Morishita, T., Sugino, O. & Otani, M. First-principles molecular dynamics at a constant electrode potential. *Phys. Rev. Lett.* **109**, 266101 (2012). 23368585 10.1103/PhysRevLett.109.266101

