# Supplementary material for: Tuned interfacial hydrogen bonds for enhanced H2 electrocatalysis kinetics on Ti2C MXenes
Source: Commun Chem. 2026 Jul 13;9:246. doi: 10.1038/s42004-026-02099-z (PMC13365831; doi:10.1038/s42004-026-02099-z)
Supplement: Supplementary file 2 — Supplementary Information [file 42004_2026_2099_MOESM2_ESM.pdf]

Supporting Information: Tuned Interfacial Hydrogen Bonds for Enhanced H_2 Electrocatalysis kinetics on Ti_2C MXenes

Lin Chen,[1*] Johanna Rosen[1*], Jonas Björk[1*]

[1] *Materials Design Division, Department of Physics, Chemistry and Biology, IFM, Linköping University, 58183, Linköping, Sweden*

E-mail: lin.chen@liu.se;johanna.rosen@liu.se;jonas.bjork@liu.se

CP-CMD simulations

The explicit water films were initially equilibrated for 10ps using TIP3P force field as implemented in the Atomic Simulation Environment (ASE).[S1] Following this initial equilibration, the water films were positioned on MXenes and subsequently underwent a further equilibration using CP-AIMD for an additional 5 ps in VASP before the CP-CMD simulations. For each CMD simulation, the free-energy gradients were evaluated from trajectories collected over 5 ps with a step size of 1e-4 Å within the slow-growth approach. For the Volmer step, the collective variable (CV) was defined as the distance between the hydrogen atom in the hydronium ion and the nearest O atom on the MXene surface. For the Heyrovsky step, the CV was defined in two stages: initially as the distance between the two hydrogen atoms ($H^+ + H^*$), and subsequently as the distance between the center of mass of the adsorbed hydrogen molecule (H_2) and the O atom on the MXene surface after H-H bond formation. We note that our simulations treat the surface O/OH coverage as constant during individual HER reaction events. This assumption is justified by timescale separation: the elementary HER steps (Volmer and Heyrovsky) occur much faster than the processes that alter overall surface termination through hydroxylation/dehydroxylation. Under steady-state operation, the surface maintains an approximately constant average coverage determined by the applied potential (as indicated by our Pourbaix analysis), even as individual catalytic turnovers occur at active sites.

Lattice constants

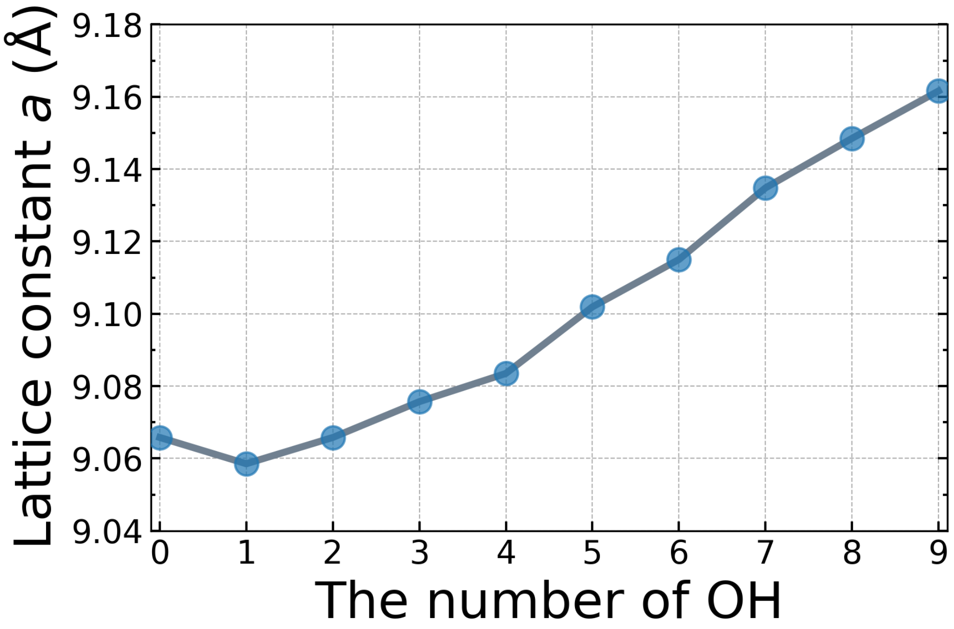

Supplementary Figure 1: The in-plane lattice constant a with respect to the number of surface termination OH. 0 and 1 denotes Ti_2C MXene fully terminated with O and OH, respectively.

Grand canonical potential energy

Supplementary Table 1. Fitted parameters for the quadratic equation for calculating the grand canonical potential energy Ω of Ti_2C MXene with various combinations of O and OH terminations. C: capacitance; U_0: potential of zero charge (PZC); E_0: the energy of the configuration at PZC.

Configurations	C(e/V)	U_0(V/SHE)	E_0(eV)
1ML O-terminated Ti_2C	0.42	1.61	-406.56
1/9ML OH-terminated Ti_2C	0.65	0.98	-414.53
2/9ML OH-terminated Ti_2C	0.67	0.47	-422.38
3/9ML OH-terminated Ti_2C	0.67	0.05	-430.03
4/9ML OH-terminated Ti_2C	0.63	-0.98	-437.14
5/9ML OH-terminated Ti_2C	0.62	-1.7	-443.91
6/9ML OH-terminated Ti_2C	0.68	-2.02	-450.31
7/9ML OH-terminated Ti_2C	0.70	-2.55	-455.94
8/9ML OH-terminated Ti_2C	0.76	-3.27	-461.87

Surface Pourbaix diagram with constant charge

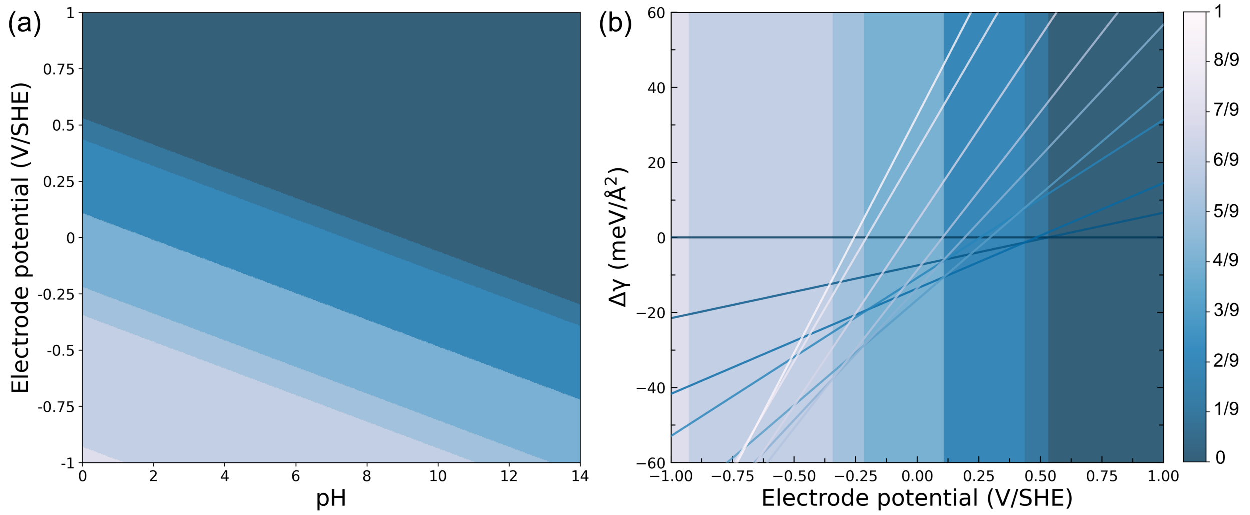

Supplementary Figure 2: (a) Surface Pourbaix diagram, *i.e.*, a map of the stable phases of Ti_2C MXene with various combinations of O and OH terminations as a function of pH and electrode potential. (b) Free energy of hydrogen adsorption per surface area on oxygen-terminated Ti_2C MXene at electrochemical interfaces as a function of electrode potential under conditions of pH 0. The color bar shows the coverage of OH species.

Constant-potential constrained MD simulations

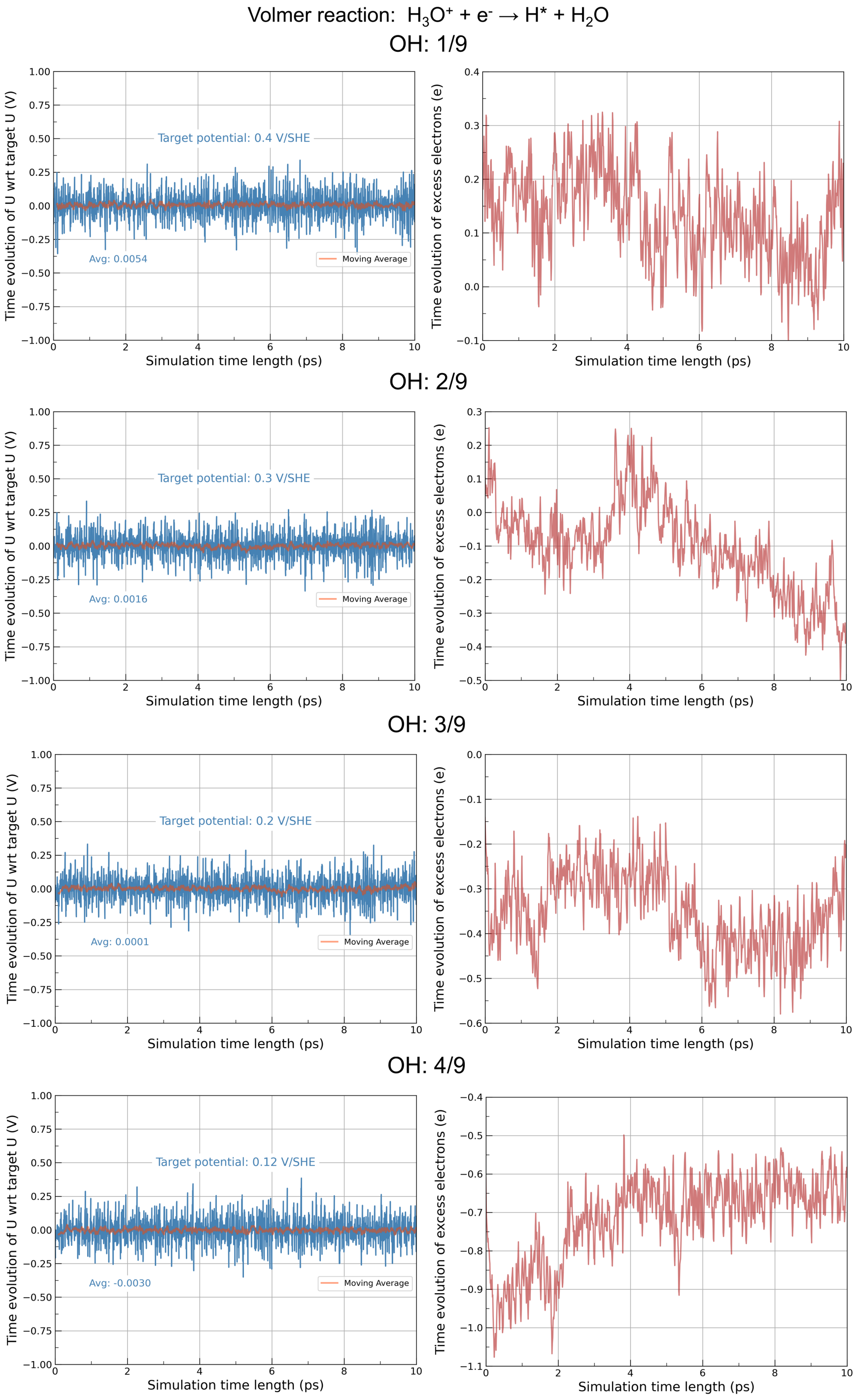

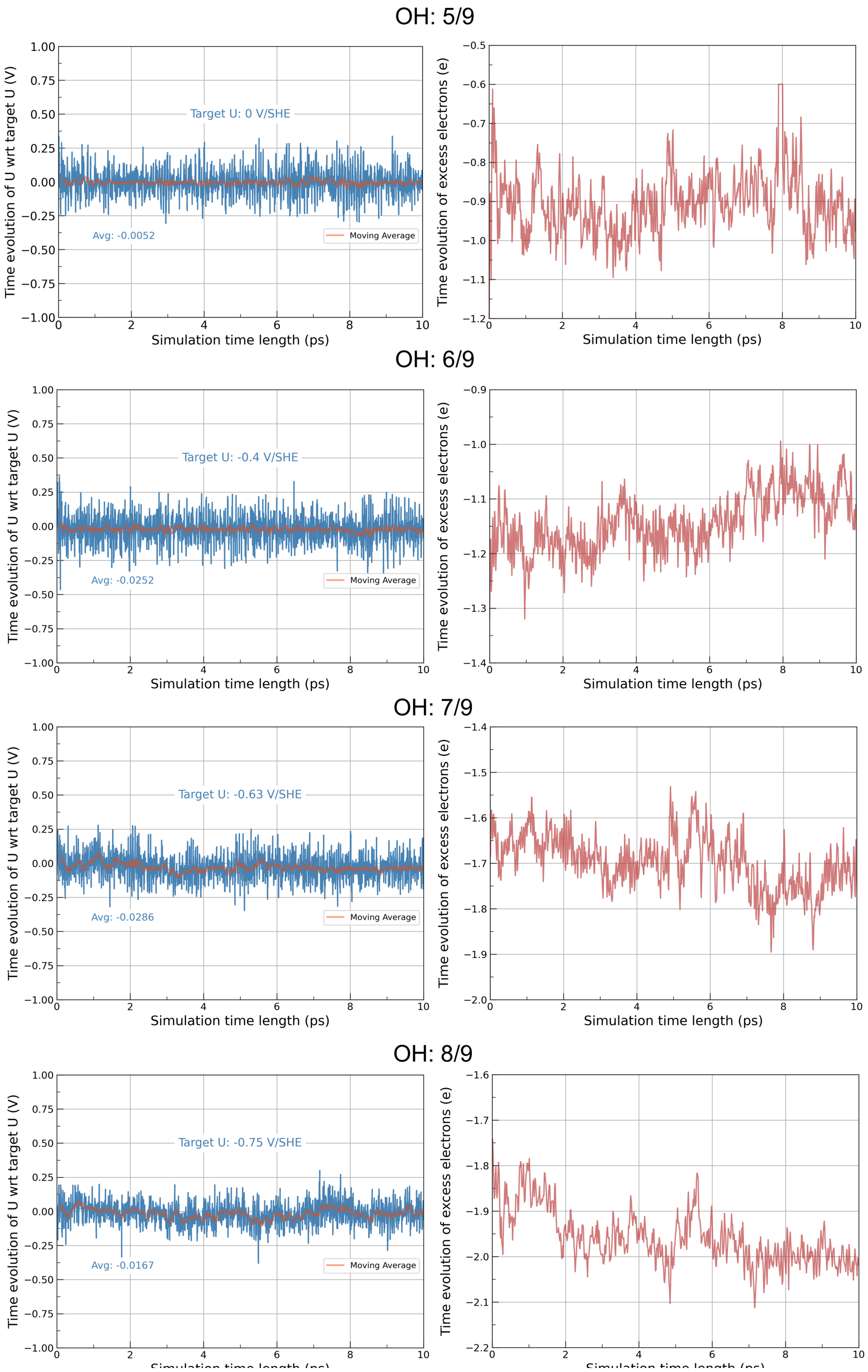

Supplementary Figure 3: The electrode potential evolved during CP-CMD simulations with respect to the target potential (blue lines) for each Ti_2C with varied coverage of OH termination. The moving average (red line) is calculated over a 100-step sliding window. The time evolution of excess electrons (pink lines) for each corresponding CP-CMD simulations are shown in the right.

Heyrovsky step on mixed O-/OH-terminated Ti_2C MXenes with OH coverage of 8/9 and on fully OH-terminated Ti_2C MXenes

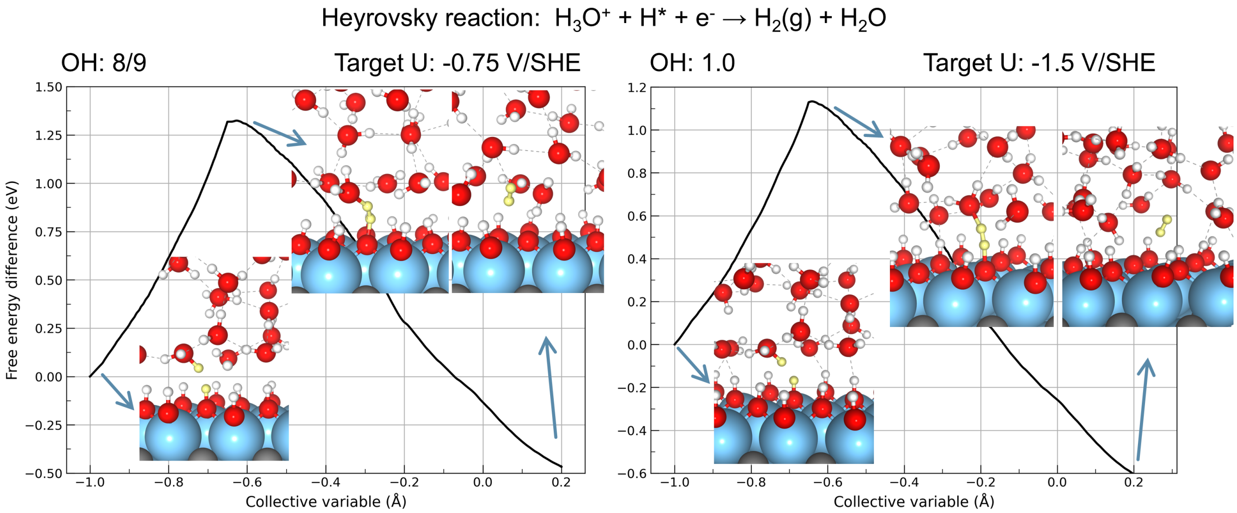

Supplementary Figure 4: Free energy profile for the Heyrovsky step on mixed O-/OH-terminated Ti_2C MXenes with OH coverage of 8/9 and on fully OH-terminated Ti_2C MXenes from constant-potential CMD simulations. Key configurations (initial, transition and final states) during constant-potential CMD simulation are highlighted with corresponding atomic structures. The atomic hydrogen participating in the PCET process is color-coded in yellow for better visual identification.

Time evolution of the interfacial distance between the bottom water layer and MXenes surface

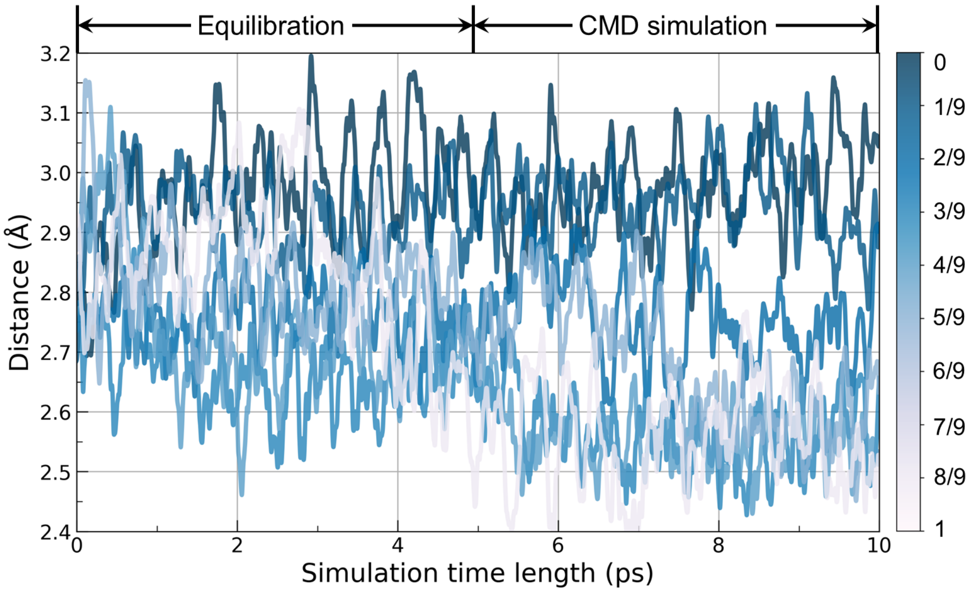

Supplementary Figure 5: Time evolution of the distance between the interfacial bottom water layer and the surface termination oxygen species on O-terminated Ti_2C MXenes and mixed O-/OH- terminated Ti_2C MXenes during constant-potential CMD simulations of the Volmer step. The entire constant-potential simulation includes 5 ps equilibration followed by 5 ps CMD simulation. The color bar shows the coverage of OH termination species.

Time evolution of the interfacial distance between the bottom water layer and O-terminated MXenes surface

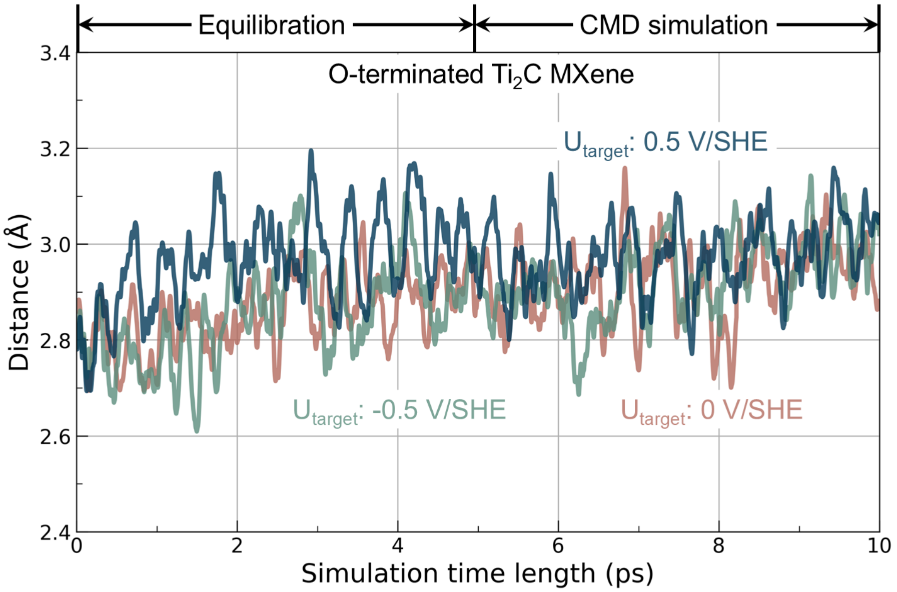

Supplementary Figure 6: Time evolution of the distance between the interfacial bottom water layer and the surface termination oxygen species on O-terminated Ti_2C MXenes during constant-potential CMD simulations of the Volmer step under the target electrode potential of 0.5 (dark blue), 0 (brown) and -0.5 (green) V/SHE, respectively. The entire constant-potential simulation includes 5 ps equilibration followed by 5 ps CMD simulation.

Zundel cation population and dynamics

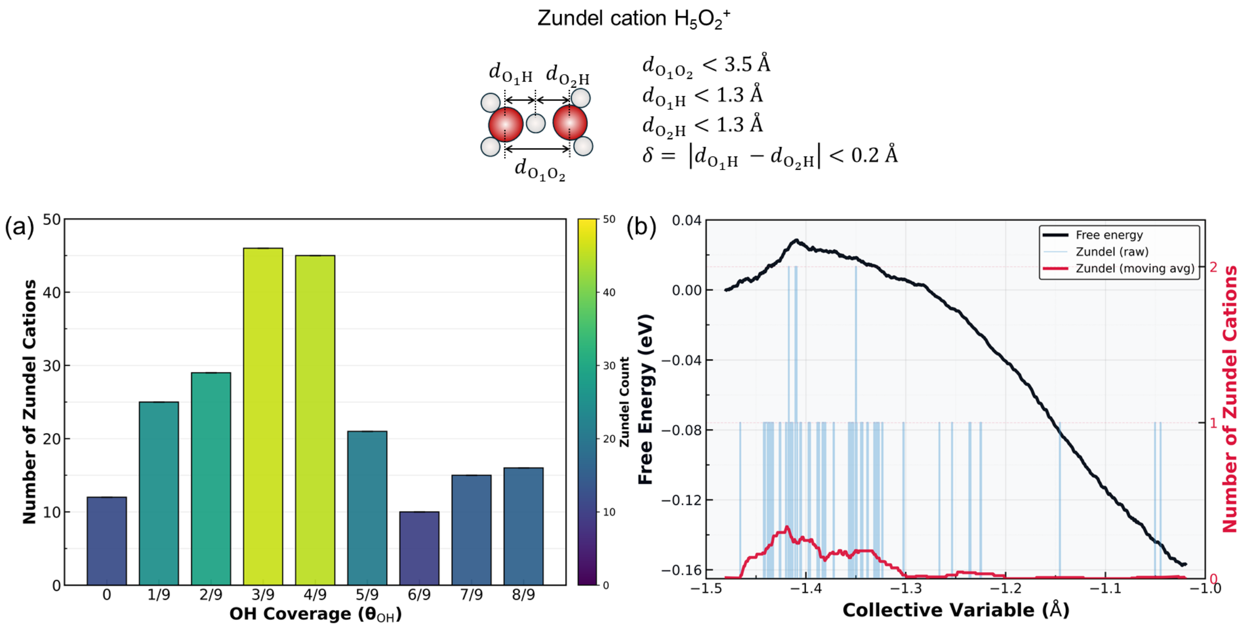

Supplementary Figure 7: (a) Number of Zundel cation occurrences in each CP-CMD trajectory of the Volmer step on pure O-terminated (0 OH coverage) and mixed O-/OH-terminated (OH coverage ranging from 1/9 to 8/9) Ti_2C MXene surfaces. (b) Cross-correlation between Zundel cation presence and the collective variable (CV) for the Volmer step on mixed O-/OH-terminated MXene with 3/9 OH coverage. The free energy profile (black curve) is overlaid for reference. Blue bars indicate individual Zundel occurrences along the trajectory, and the red curve shows the moving average calculated with a 1 ps window. The geometric criteria for identifying Zundel cations are shown schematically above the figures.

Charge density difference

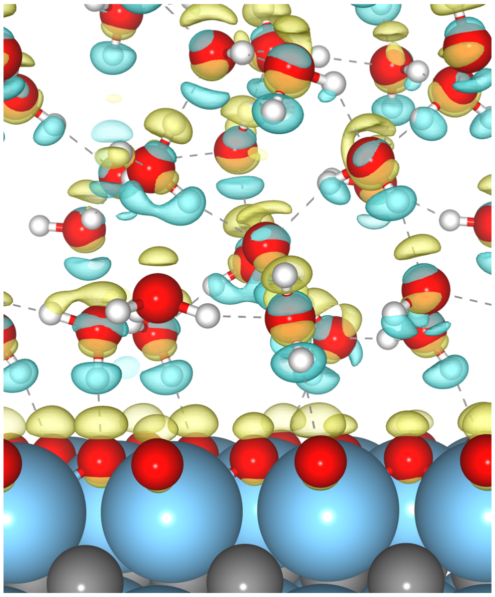

Supplementary Figure 8: Charge density difference between the initial structure for the Volmer step on O-terminated Ti_2C MXene/water interface with and without external electric field. The electrode potential is set to 0.5 V/SHE. Yellow isosurfaces indicate electron accumulation and blue isosurfaces indicate electron depletion. The isosurface level is set to 3e-4 e/Bohr^3.

Radial distribution function analysis

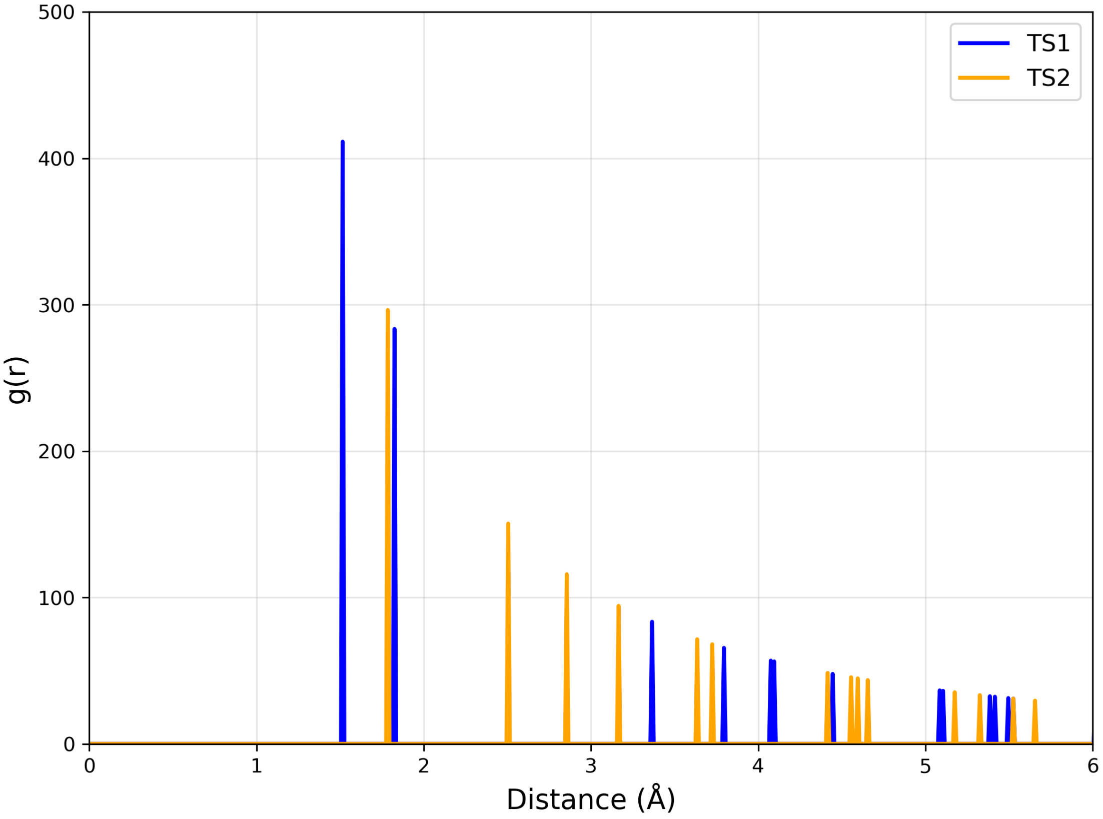

Supplementary Figure 9: Radial distribution functions g(r) calculated at the two transition states. The radial distribution function is calculated between the center of mass of the two reacting hydrogen atoms and the oxygen atoms of interfacial water molecules.

References

(S1) Larsen, A. H.; Mortensen, J. J.; Blomqvist, J.; Castelli, I. E.; Christensen, R.; Dułak, M.; Friis, J.; Groves, M. N.; Hammer, B.; Hargus, C.; others *Journal of Physics: Condensed Matter* **2017**, *29*, 273002.
