# Supplementary material for: Tuned interfacial hydrogen bonds for enhanced H2 electrocatalysis kinetics on Ti2C MXenes
Source: Commun Chem. 2026 Jul 13;9:246. doi: 10.1038/s42004-026-02099-z (PMC13365831; doi:10.1038/s42004-026-02099-z)
Supplement: Supplementary file 1 — Transparent Peer Review file [file 42004_2026_2099_MOESM1_ESM.pdf]

nature portfolio

# Peer Review File

# Tuned Interfacial Hydrogen Bonds for Enhanced $H_2$ Electrocatalysis kinetics on $Ti_2C$ MXenes

Corresponding Author: Dr Jonas Björk

**This file contains all reviewer reports in order by version, followed by all author rebuttals in order by version.**

Version 0:

Reviewer comments:

Reviewer #1

(Remarks to the Author)
By means of constant-potential constrained molecular dynamics simulations, this manuscript reveals that introducing OH groups onto Ti2C MXene surfaces reconstructs the hydrogen-bond network and markedly accelerates acidic hydrogen evolution kinetics. Mixed O/OH-terminated surfaces are thermodynamically stable under negative potentials, combining high activity with durability. It offers an atomistic strategy for engineering electrocatalyst interfaces. Despite the contribution, some issues should be addressed before further consideration.
(1) Provide data for the fully OH-terminated surface (Fig. S4 only shows Heyrovsky at 8/9 coverage) to cover all termination types completely.
(2) The authors claim that the “stabilization” of the Zundel intermediate lowers the barrier, yet the CP-CMD trajectories contain no analysis of its continuous lifetime or population. Please provide the Zundel-Eigen interconversion rate and the cross-correlation function between this rate and the collective variable of the Volmer step, to establish a direct causal link: “stabilized Zundel→reduced activation energy”.
(3) Several articles “the” are missing, and verb tenses are mixed; a full-text proofread is recommended.
(4) The surface functional group of the MXene (e.g., -O, -OH, -Cl, and -F) plays an important role in the alkaline hydrogen evolution reaction (Acta Materialia 283, 120507 (2025)). By combining the progresses, the authors are encouraged to discuss whether the surface hydroxyl groups on Ti2C MXenes remain effective across a wide pH range.
(5) Under constant-potential conditions, the total electron count varies with the reaction coordinate; Figure 3b shows fluctuations as large as 0.6 e-. Are these excess electrons localized mainly in Ti 3d states, in the adsorbed H 1s orbital, or delocalized over interfacial water? Please present the projected density of states (PDOS) or a stepwise Bader-charge analysis to clarify whether “electron injection” alters the Ti valence state and indirectly modulates H adsorption strength, rather than acting solely through the hydrogen-bond network.
(6) In the Methods section, explicitly state the O-O and O-H distance thresholds and angle cutoff used for the hydrogen-bond criterion.

Reviewer #2

(Remarks to the Author)
This work employs constant-potential constrained molecular dynamics simulations to investigate the influence of surface termination (O/OH) on hydrogen evolution reaction kinetics in Ti2C MXenes. It proposes hydrogen bond network connectivity as a novel kinetic descriptor, transcending the traditional ΔGH framework and demonstrating significant theoretical innovation. Integrating surface Pourbaix analysis, dynamic interface simulations, and free energy calculations, this work demonstrates high methodological integration, offering novel perspectives for electrocatalytic interface design. However, experimental validation remains limited, and discussions on the universality of simulation results are insufficient. It is recommended to incorporate additional control conditions to enhance the reliability of conclusions.

1. This work provides only a brief description of the detailed parameters for the CP-CMD simulation (e.g., time step). It is recommended to include more technical details in the Methods section to ensure reproducibility.

2. Figure 3a shows that the electrode potential fluctuates near the target value with an average deviation close to zero, demonstrating the robustness of the method. However, the fluctuation range is relatively large. How does this fluctuation

affect the reliability of the results?

3. The manuscript reports that the excess electron density in the system varies between 0.1 and 0.6 e, but does not discuss the long-term impact of this variation on the interface structure. It is recommended to analyze the correlation between electron fluctuations and interface reconstruction.

4.Figure 4c shows that the Heyrovsky step possesses two transition states with a barrier as high as 1.7 eV. However, the document does not explain why the H2 desorption barrier is lower. It is recommended to analyze the role of the interfacial water network during the desorption process.

2. The free energy profile in Figure 5 only shows cases with OH coverage ranging from 1/9 to 8/9, but does not include a comparison with pure OH termination (9/9). It is recommended to supplement this to comprehensively evaluate the trend.

6. Figure 7a demonstrates that mixing termination shortens the water-MXene interfacial distance. However, the time evolution diagram only illustrates the 4/9 OH coverage example, and data for other coverages are not sufficiently discussed in the manuscript. It is recommended to consolidate the primary findings.

7. The conclusion highlights the enhanced effect of hybrid termination, but the requirement for negative potential to achieve high OH coverage in the documentation may limit practical application. It is recommended to mention the potential-dependent challenges in the conclusion.

Reviewer #3

(Remarks to the Author)
This study provides a systematic investigation into the influence of surface terminations (O/OH) in $Ti_2C$ MXene on the interfacial hydrogen-bond network and hydrogen evolution reaction kinetics. The proposed concept of "hydrogen-bond network connectivity" offers an innovative extension beyond the conventional ΔGH descriptor. Nevertheless, several key aspects of the work require further substantiation to strengthen its conclusions.

1．The total simulation duration of 10 ps is reported for the MD studies. However, whether this timescale is sufficient for the system to attain complete equilibrium remains inadequately justified.

2. The study employs a solvation model with four explicit water layers (approximately 30 molecules). It is important to assess whether this model size is sufficient to capture the long-range hydrogen-bond networks relevant to the proton transfer mechanism under investigation.

3．The study assumes that the surface O/OH coverage remains constant during the reaction. However, under realistic electrocatalytic conditions, the electrode surface may undergo dynamic changes, such as the adsorption and desorption of OH species. It is necessary to discuss whether this assumption constitutes a limitation of the model.

4. The physical-chemical origin of how OH terminations reduce the interfacial water-MXene separation remains unclear. Please provide further analysis to elucidate the fundamental mechanism behind this structural effect.

5. The novelty of this work should be more clearly articulated through explicit comparison with existing theoretical studies, highlighting the distinct insights or advancements this study provides.

Version 1:

Reviewer comments:

Reviewer #1

(Remarks to the Author)
The authors have adequately addressed all of my concerns. I recommend the manuscript for publication.

Reviewer #2

(Remarks to the Author)
hanks to the author for the point-by-point response to the comments. I agree to accept this manuscript.

Reviewer #3

(Remarks to the Author)
The authors revised the manuscript well based on the comments, it can be published now

**Open Access** This Peer Review File is licensed under a Creative Commons Attribution 4.0 International License, which permits use, sharing, adaptation, distribution and reproduction in any medium or format, as long as you give appropriate credit to the original author(s) and the source, provide a link to the Creative Commons license, and indicate if changes were made.
In cases where reviewers are anonymous, credit should be given to 'Anonymous Referee' and the source.
The images or other third party material in this Peer Review File are included in the article's Creative Commons license, unless indicated otherwise in a credit line to the material. If material is not included in the article's Creative Commons license and your intended use is not permitted by statutory regulation or exceeds the permitted use, you will need to obtain permission directly from the copyright holder.
To view a copy of this license, visit https://creativecommons.org/licenses/by/4.0/

We thank the Reviewers for their time and comments, which have helped us to improve the manuscript. A point-by-point response to the comments are given below. The comments are in black, and our responses are in blue.

Reviewers' comments:

**Reviewer #1 (Remarks to the Author):**

By means of constant-potential constrained molecular dynamics simulations, this manuscript reveals that introducing OH groups onto Ti2C MXene surfaces reconstructs the hydrogen-bond network and markedly accelerates acidic hydrogen evolution kinetics. Mixed O/OH-terminated surfaces are thermodynamically stable under negative potentials, combining high activity with durability. It offers an atomistic strategy for engineering electrocatalyst interfaces. Despite the contribution, some issues should be addressed before further consideration.

(1) Provide data for the fully OH-terminated surface (Fig. S4 only shows Heyrovsky at 8/9 coverage) to cover all termination types completely.

Reply: We thank the reviewer for this valuable suggestion. We have performed additional constant-potential CMD simulations for the Heyrovsky step on fully OH-terminated $Ti_2C$ MXene ($\theta_{OH} = 1.0$). These simulations were carried out using the same computational protocol as the mixed-termination cases, enabling a direct and consistent comparison. The resulting free energy profile (below Figure 1) is now included in Figure S4 in the revised Supplementary Information.

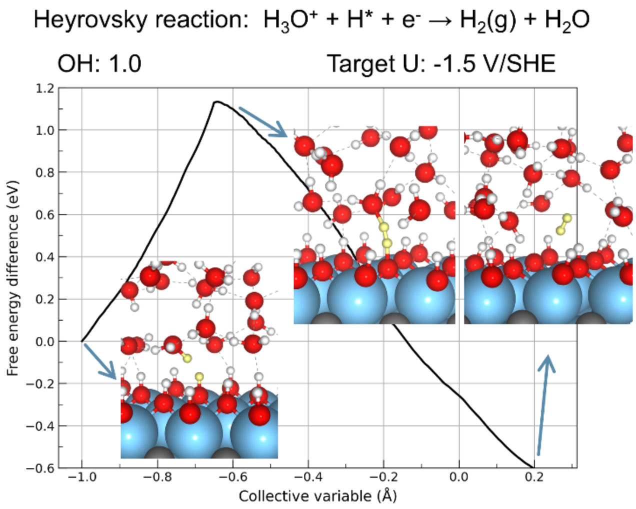


Figure 1 Free energy profile for the Heyrovsky step on fully OH-terminated $Ti_2C$ MXenes from constant-potential CMD simulations. Key configurations (initial, transition and final states) during constant-potential CMD simulation are highlighted with corresponding atomic structures. The atomic hydrogen participating in the PCET process is color-coded in yellow for better visual identification.

The fully OH-terminated surface exhibits a single activation barrier of approximately 1.1 eV, which is lower than those on pure O-terminated and mixed O/OH-terminated surfaces, consistent with the trend of decreasing barrier with increasing OH coverage. The transition state structure again involves a proton-coupled electron transfer event. Molecular hydrogen desorption is barrierless and strongly exergonic, as observed for all coverage cases. These new results have been incorporated into the revised Figure S4 and are described in the Results section on Page 17 of the manuscript.

(2) The authors claim that the "stabilization" of the Zundel intermediate lowers the barrier, yet the CP-CMD trajectories contain no analysis of its continuous lifetime or population. Please provide the Zundel-Eigen interconversion rate and the cross-correlation function between this rate and the collective variable of the Volmer step, to establish a direct causal link: "stabilized Zundel→reduced activation energy".

Reply: We agree that a more detailed kinetic analysis of the Zundel cation intermediate is necessary to establish a direct causal link between its stabilization and the reduction of the activation energy for the Volmer step. In response to the reviewer's comment, we have performed the following additional analyses on our CP-CMD trajectories:

1. Zundel cation population analysis: We quantified the occurrence frequency of Zundel cation along the trajectory for both pure O-terminated and mixed O/OH-terminated MXene surfaces across a range of OH coverages (below Figure 2(a)). The Zundel cation is identified using geometric criteria based on O-O and O-H distances, as illustrated schematically at the top of Figure 2.

The results reveal a non-monotonic dependence of Zundel population on OH coverage. Specifically, Zundel cations appear most frequently at intermediate OH coverages, with the 3/9 (1/3) coverage exhibiting the highest occurrence. As OH coverage increases further, Zundel occurrences decrease, which we attribute to earlier onset of proton-coupled electron transfer (PCET) along the reaction coordinate. This earlier PCET manifests as a more facile, nearly barrierless hydrogen adsorption process at higher OH coverages, wherein the proton transfer becomes sufficiently favorable that the system bypasses the need for extended Zundel stabilization.

2. Cross-correlation between Zundel presence and the collective variable (CV) for the Volmer step: We analyzed the distribution of Zundel cation occurrences as a function of the CV for the Volmer step (below Figure 2(b)), focusing on the mixed O-/OH-terminated surface with 3/9 OH coverage where Zundel formation is most prominent. This analysis directly addresses whether Zundel formation is temporally and mechanistically correlated with the proton approaching the transition state.

The results demonstrate that Zundel cation formation occurs preferentially in the vicinity of the transition state region, with occurrences predominantly localized around the barrier. The moving average (calculated using a 1000-step window) shows a peak slightly preceding the transition state position along the CV. This temporal correlation

provides direct evidence that Zundel cation formation facilitates the transition state crossing by pre-organizing the proton-transfer pathway. The Zundel intermediate effectively facilitates proton transfer by reducing the reorganization energy required for the proton to move from the hydronium ion to the surface. Our CP-CMD simulations reveal a consistent mechanistic pathway involving Zundel-mediated proton transfer at the MXene/water interface, which becomes increasingly favorable with appropriate OH functionalization.

The corresponding analysis and results have been added to the Supporting Information as Figure S7, with discussion included in the revised manuscript on Page 18-19.

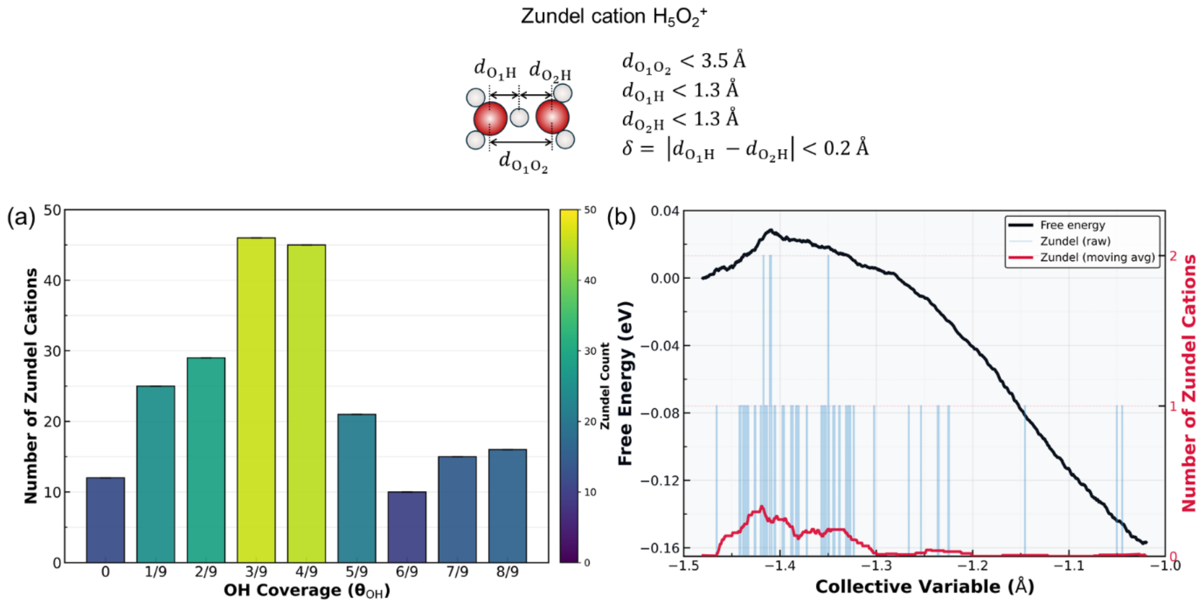


Figure 2: (a) Number of Zundel cation occurrences in each CP-CMD trajectory of the Volmer step on pure O-terminated (0 OH coverage) and mixed O-/OH-terminated (OH coverage ranging from 1/9 to 8/9) $Ti_2C$ MXene surfaces. (b) Cross-correlation between Zundel cation presence and the collective variable (CV) for the Volmer step on mixed O-/OH-terminated MXene with 3/9 OH coverage. The free energy profile (black curve) is overlaid for reference. Blue bars indicate individual Zundel occurrences along the trajectory, and the red curve shows the moving average calculated with a 1 ps window. The geometric criteria for identifying Zundel cations are shown schematically above the figures.

(3) Several articles "the" are missing, and verb tenses are mixed; a full-text proofread is recommended.

Reply: We thank the reviewer for this careful observation. We have conducted a thorough proofread of the entire manuscript to correct missing articles and ensure consistent verb tense usage throughout. The revised manuscript has been carefully reviewed for grammatical accuracy and clarity.

(4) The surface functional group of the MXene (e.g., -O, -OH, -Cl, and -F) plays an important role in the alkaline hydrogen evolution reaction (Acta Materialia 283, 120507 (2025)). By combining the progresses, the authors are encouraged to discuss whether the surface hydroxyl groups on Ti2C MXenes remain effective across a wide pH range.

Reply: We thank the reviewer for directing us to the recent work on “MXene quantum dots-$Co(OH)_2$ heterojunction stimulated $Co^{2+\delta}$ sites for boosted alkaline hydrogen evolution” (Acta Materialia 283, 120507 (2025)), which provides valuable insights into alkaline HER mechanisms that complement our acidic HER study.

In the alkaline HER study, the authors demonstrate that electron-rich MXene quantum dots facilitate $H_2O$ molecule adsorption onto the electrode surface. This enhanced water-electrode interaction promotes the subsequent $H_2O$ dissociation on $Co^{2+\delta}$ site, with the improved interfacial contact ensuring efficient electron transfer and accelerating the kinetically hindered $H_2O$ dissociation step.

Our findings show a parallel effect in acidic conditions. Our CP-CMD simulations show that surface OH groups serve as structural anchors that reorganize the interfacial water network within the electric double layer, transforming discontinuous H-bond arrangements into interconnected H-bond networks. This enhanced interfacial connectivity facilitates proton transfer from hydronium ions to the surface.

Both cases demonstrate that appropriate surface functionalization breaks down the interfacial barrier between the electrode and aqueous electrolyte, creating a more favorable environment for the subsequent steps. The underlying commonality is interfacial optimization: enhanced water adsorption promotes $H_2O$ dissociation in alkaline media, while enhanced H-bond connectivity facilitates proton transfer in acidic media.

We have added a brief discussion of these pH-dependent considerations in the revised manuscript (Page 22).

(5) Under constant-potential conditions, the total electron count varies with the reaction coordinate; Figure 3b shows fluctuations as large as 0.6 e-. Are these excess electrons localized mainly in Ti 3d states, in the adsorbed H 1s orbital, or delocalized over interfacial water? Please present the projected density of states (PDOS) or a stepwise Bader-charge analysis to clarify whether “electron injection” alters the Ti valence state and indirectly modulates H adsorption strength, rather than acting solely through the hydrogen-bond network.

Reply: We thank the reviewer for the comment regarding the nature of electron injection and charge localization under constant-potential conditions. To address this question, we have performed charge density difference analysis to elucidate the spatial distribution of excess electrons.

We calculated the charge density difference between the initial structure for the Volmer step on O-terminated $Ti_2C$ MXene/water interface with and without external electric field, defined as:

$$\Delta\rho = \rho(\text{with external electric field}) - \rho(\text{without external electric field})$$

where $\rho(\text{with external electric field})$ is the charge density of the structure under the target electrode potential of 0.5 V/SHE and $\rho(\text{without external electric field})$ is the charge density of the same structure without external electric field.

The charge density difference (below Figure 3) reveals that the excess electrons delocalized over both the surface oxygen atoms and the interfacial water network. Electron accumulation (yellow/positive isosurface) is observed on the surface O-termination sites and extends into the oxygen atoms of water molecules in the interfacial region. Simultaneously, electron depletion (blue/negative isosurface) occurs on H atoms in the interfacial water molecules. Thus, the injected electrons are accommodated predominantly by the surface oxygen sites and distributed throughout the interfacial water network.

These results demonstrate that the electron injection mechanism operates primarily through electron spillover into the interfacial water layer. The delocalized electron density enhances the overall electrostatic environment of the interfacial region and strengthens the hydrogen bonding network. This electron spillover into the water network partially screens the positive charge of the approaching hydronium ion and stabilizes negatively charged oxygen sites, thereby reducing the electrostatic repulsion and reorganization energy required for proton transfer. Consequently, the activation barrier for the Volmer step is lowered.

This charge density analysis has been added to the Supporting Information (Figure S8), and the mechanistic discussion has been incorporated into the revised manuscript (Page 10-11).

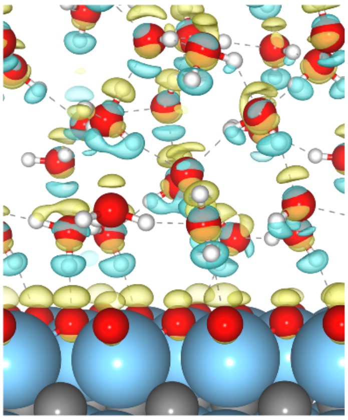

Figure 3 Charge density difference between the initial structure for the Volmer step on O-terminated $Ti_2C$ MXene/water interface with and without external electric field. The electrode potential is set to 0.5 V/SHE. Yellow isosurfaces indicate electron accumulation and blue isosurfaces indicate electron depletion. The isosurface level is set to 3e-4 e/$Bohr^3$.

(6) In the Methods section, explicitly state the O-O and O-H distance thresholds and angle cutoff used for the hydrogen-bond criterion.

Reply: We have now explicitly stated the geometric criteria used to define hydrogen bonds in the revised Methods section. The following details have been added to the manuscript:

"Hydrogen bonds were defined using the following geometric criteria: O···O distance ⩽ 3.5 Å, 1.2 Å ⩽ H···O distance ⩽ 2.1 Å, and O-H···O angle ⩾ 135°."

**Reviewer #2 (Remarks to the Author):**

This work employs constant-potential constrained molecular dynamics simulations to investigate the influence of surface termination (O/OH) on hydrogen evolution reaction kinetics in Ti2C MXenes. It proposes hydrogen bond network connectivity as a novel kinetic descriptor, transcending the traditional ΔGH framework and demonstrating significant theoretical innovation. Integrating surface Pourbaix analysis, dynamic interface simulations, and free energy calculations, this work demonstrates high methodological integration, offering novel perspectives for electrocatalytic interface design. However, experimental validation remains limited, and discussions on the universality of simulation results are insufficient. It is recommended to incorporate additional control conditions to enhance the reliability of conclusions.

Reply: We are delighted that the reviewer acknowledges the novel aspects of our work.

1. This work provides only a brief description of the detailed parameters for the CP-CMD simulation (e.g., time step). It is recommended to include more technical details in the Methods section to ensure reproducibility.

Reply: More technical details have been included in the Supporting Information under the section of CP-CMD simulations. We have also revised the corresponding text on Page 27 to direct readers to the Supporting Information for these details.

2. Figure 3a shows that the electrode potential fluctuates near the target value with an average deviation close to zero, demonstrating the robustness of the method. However, the fluctuation range is relatively large. How does this fluctuation affect the reliability of the results?

Reply: We thank the reviewer for this important question regarding the potential fluctuations observed in our CP-CMD simulations. The fluctuation range, while visually apparent, is consistent with the expected physical behavior of electrochemical interfaces and does not compromise the reliability of our results. We address this concern from several perspectives:

**1) Physical origin of fluctuations**. The potential fluctuations arise from the fundamental nature of the grand-canonical ensemble implementation, where the number of electrons fluctuates to maintain a constant electrochemical potential. These fluctuations reflect the dynamic charge transfer processes occurring at the electrified interface—as the proton approaches the surface during the Volmer step, the local electrostatic environment changes, requiring instantaneous adjustments in the electrode charge to maintain the target potential. The observed fluctuation range within

±0.25 V around the target potential is physically reasonable and comparable to thermal fluctuations at electrochemical interfaces under realistic conditions.

**2) Statistical validity**. The robustness of the CP-CMD method is demonstrated by the near-zero mean deviation (-0.0019 V) of the electrode potential from the target value over the entire trajectory. This small time-averaged deviation indicates that the system is properly equilibrated around the target potential. The free energy gradients used in thermodynamic integration are calculated as ensemble averages over these fluctuating trajectories, which properly capture the statistical mechanics of the constant-potential ensemble. The fluctuations themselves are part of the physical sampling that ensures proper thermal averaging.

**3) Comparison with constant-charge simulations.** The critical comparison is not the absolute magnitude of fluctuations in CP-CMD, but rather the difference between CP-CMD and constant-charge approaches. As shown in Figure 3a, the constant-charge simulation exhibits systematic deviations up to +0.9 V from the target potential during the reaction and represents unphysical drift rather than equilibrium fluctuations. The CP-CMD fluctuations are centered around the target potential with zero mean drift, whereas constant-charge simulations show systematic potential shifts that do not represent the intended electrochemical conditions.

**4) Impact on free energy profiles.** The thermodynamic integration method inherently accounts for these fluctuations by averaging over the ensemble of configurations sampled during each constrained MD window. The relatively smooth free energy profiles obtained without unphysical discontinuities demonstrate that the fluctuations do not introduce significant noise into the final free energy estimates.

In summary, the potential fluctuations observed in our CP-CMD simulations are: (1) physically meaningful representations of charge transfer dynamics at electrochemical interfaces, (2) properly controlled around the target potential with negligible mean deviation, (3) significantly smaller than the systematic errors in constant-charge approaches, and (4) properly accounted for through ensemble averaging in the thermodynamic integration procedure. Besides, **our observed fluctuation range is consistent with other CP-CMD studies reported in the literature** (*J. Am. Chem. Soc., 2022, 144, 17140*; *Nat. Commun., 2024, 15, 10099*). In our simulations, the mean deviation from the target potential is small, and the resulting free energy profiles are smooth. We believe that these observations suggest reasonable control of the electrochemical conditions.

3. The manuscript reports that the excess electron density in the system varies between 0.1 and 0.6 e, but does not discuss the long-term impact of this variation on the interface structure. It is recommended to analyze the correlation between electron fluctuations and interface reconstruction.

Reply: We thank the reviewer for this suggestion regarding the relationship between electron fluctuations and interfacial structure. To address this concern, we have analyzed how the variation in excess electrons (0.1-0.6 e) influences the interfacial configuration. We performed charge density difference analysis to elucidate the spatial distribution of excess electrons (Figure S8).

The charge density difference between the initial structure for the Volmer step on O-terminated $Ti_2C$ MXene/water interface with and without external electric field is defined as:

$$\Delta\rho = \rho(\text{with external electric field}) - \rho(\text{without external electric field})$$

where $\rho(\text{with external electric field})$ is the charge density of the structure under the target electrode potential of 0.5 V/SHE and $\rho(\text{without external electric field})$ is the charge density of the same structure without external electric field. The results show that the excess electrons are primarily delocalized over the surface oxygen atoms and the interfacial water network, with minimal localization on Ti atoms. This delocalization pattern indicates that electron fluctuations primarily modulate the electrostatic environment of the interfacial region rather than inducing significant structural reconstruction of the MXene surface itself.

To quantify the impact of electron fluctuations on interface structure, we analyzed the correlation between instantaneous excess electron count and MXene-water interfacial distance (below Figure 4). MXene-water interfacial distance was defined by the distance between the MXene surface oxygen atoms and the first water layer. The analysis shows that this interfacial gap remains stable at 2.98±0.18 Å throughout the

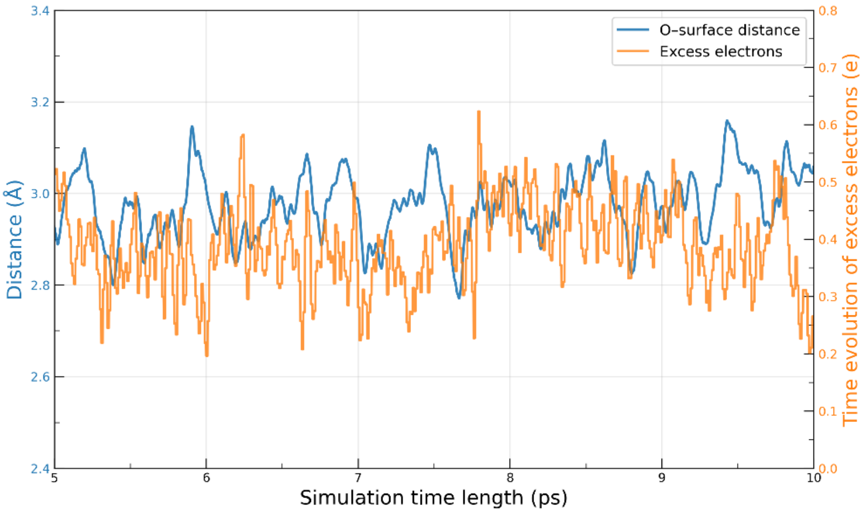


Figure 4 Time evolution of MXene-water interfacial distance (blue line) and excess electrons (orange line) during the CP-CMD simulation (without the equalibration period).

simulation, with variations of less than 0.18 Å despite the 0.6 e fluctuation in electron count. This indicates that the overall interfacial separation is not significantly affected by electron fluctuations.

The relative insensitivity of interfacial structure to electron fluctuations can be understood from two perspectives: First, the electron fluctuations (0.1-0.6 e) are distributed over an extended interfacial region encompassing multiple surface O atoms and water molecules, rather than being localized at a single site. This delocalization dilutes the structural impact at any given location. Second, the timescale of electron fluctuations (~fs) is comparable to or faster than the timescale of significant structural rearrangements of the H-bond network. The interfacial structure responds to the electrostatic environment rather than to instantaneous electron count fluctuations. Since the mean excess electron count is relatively stable over longer timescales (as reflected by the near-zero mean potential deviation), the time-averaged interfacial structure remains well-defined.

4.Figure 4c shows that the Heyrovsky step possesses two transition states with a barrier as high as 1.7 eV. However, the document does not explain why the H2 desorption barrier is lower. It is recommended to analyze the role of the interfacial water network during the desorption process.

Reply: We thank the reviewer for the question about the $H_2$ desorption barrier. The first transition state (PCET) has a barrier of 1.7 eV and the second transition state ($H_2$ desorption) shows a lower barrier of 0.5 eV. We have analyzed the role of the interfacial water network during the desorption process to explain this difference.

To understand why $H_2$ desorption has a lower barrier despite following the high-energy PCET step, we calculated the radial distribution functions (RDFs) between the reacting species and interfacial water molecules at the two transition states (below Figure 5). We characterized the solvation environment by calculating the radial distribution function (RDF) between the center of mass of the two reacting hydrogen atoms and the oxygen atoms of interfacial water molecules.

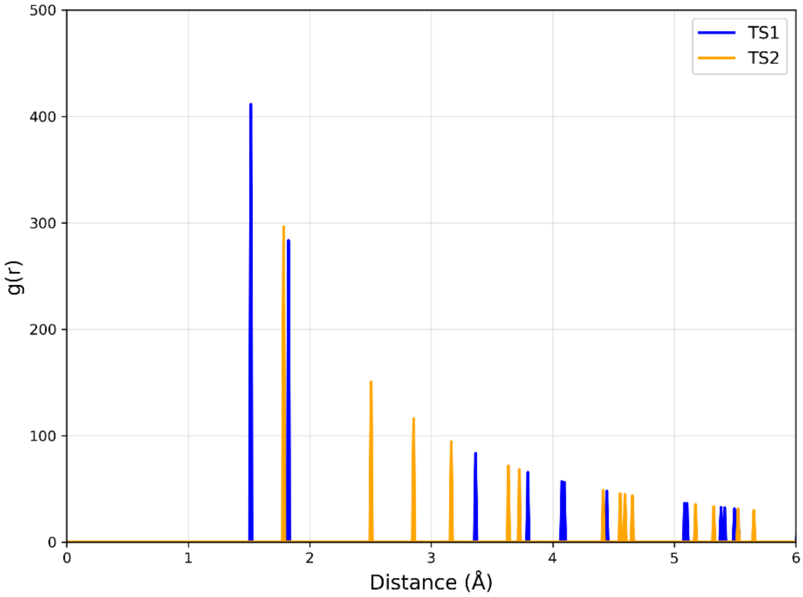


*Figure 5 Radial distribution functions g(r) calculated at the two transition states during the Heyrovsky step. The radial distribution function is calculated between the center of mass of the two reacting hydrogen atoms and the oxygen atoms of interfacial water molecules.*

At first transition state, one hydrogen is part of the hydronium ion ($H_3O^+$) while the other is adsorbed on the surface (H*), and they are approaching each other to form the H-H bond. At the second transition state ($H_2$ desorption), the character of the reacting species has changed fundamentally from charged/polar ($H_3O^+$ + H*) to neutral and nonpolar ($H_2$). The RDF analysis shows a stronger solvation of the reacting hydrogen pair by interfacial water molecules at TS1 (blue line) than TS2 (orange line). The strong solvation shell at TS1 reflects the fact that the hydronium-bound hydrogen is still participating in the extensive hydrogen bond network of $H_3O^+$. The RDF for TS2 shows a weakened first peak and an overall flattened distribution, indicating much weaker interaction between the $H_2$ molecule and the interfacial water network. The neutral $H_2$ molecule forms only weak van der Waals interactions with surrounding water molecules, whereas the charged hydronium ion participates in strong directional hydrogen bonding. Water molecules can readily accommodate the departure of neutral $H_2$ with minimal reorganization because there are no strong H-bonds to break. The lower desorption barrier at TS2 primarily arises from breaking weak $H_2$-surface physisorption rather than from H-bond reorganization.

This analysis explains why the desorption barrier is relatively low despite the overall strongly endergonic nature of the reaction. The energetic cost is front-loaded into the PCET step where extensive water reorganization is required, while the subsequent desorption benefits from the dramatically reduced interaction strength between neutral $H_2$ and both the surface and the interfacial water network.

We have added this structural analysis to the revised manuscript (Page 11, Page14) and Supporting Information (Figure S9).

5. The free energy profile in Figure 5 only shows cases with OH coverage ranging from 1/9 to 8/9, but does not include a comparison with pure OH termination (9/9). It is recommended to supplement this to comprehensively evaluate the trend.

Reply: We thank the reviewer for this suggestion. We would like to clarify the rationale for not including pure OH termination (9/9 coverage) in the Volmer step analysis shown in Figure 5, while noting that we have indeed performed Heyrovsky step calculations at 9/9 OH coverage as shown in Figure S4.

The Volmer step involves proton transfer from hydronium ion ($H_3O^+$) to the MXene surface with concurrent electron transfer, producing an adsorbed hydrogen atom (H*). This reaction requires an available surface oxygen site to accommodate the adsorbed hydrogen. At 9/9 OH coverage, all surface sites are already occupied by hydroxyl groups, leaving no available O sites for H adsorption. Therefore, the Volmer step as defined in our study is not feasible on fully OH-terminated surfaces, and including this coverage would not provide meaningful comparison for this particular reaction step.

We recognize the value of exploring the upper limit of OH functionalization. To complete the coverage series, we have performed CP-CMD simulations of the Heyrovsky step on 9/9 OH coverage surfaces, with results presented in Figure S4. These calculations demonstrate that the trend of decreasing barrier with increasing OH coverage continues up to full OH termination. The fully OH-terminated surface (9/9) provides the most favorable pathway for the Heyrovsky step among all coverages investigated, consistent with the monotonic trend observed across the coverage series. This result is mentioned in the manuscript on Page 17.

6. Figure 7a demonstrates that mixing termination shortens the water-MXene interfacial distance. However, the time evolution diagram only illustrates the 4/9 OH coverage example, and data for other coverages are not sufficiently discussed in the manuscript. It is recommended to consolidate the primary findings.

Reply: While we provided the complete interfacial distance data for all coverages in Figure S5, we agree that the coverage-dependent trends deserve more explicit discussion in the main manuscript. We have expanded the discussion on Page 18-19 as follows:

"As shown in Figure S5, the time-evolved interfacial distances are color-coded by coverage, revealing a systematic downward shift with increasing OH coverage despite thermal fluctuations in individual trajectories. This progressive reduction demonstrates that surface OH groups consistently bring the interfacial water layer closer to the MXene surface, enhancing H-bond network connectivity."

7. The conclusion highlights the enhanced effect of hybrid termination, but the requirement for negative potential to achieve high OH coverage in the documentation

may limit practical application. It is recommended to mention the potential-dependent challenges in the conclusion.

Reply: We thank the reviewer for this point regarding the practical implications of our findings. We agree that discussing the potential-dependent stability of OH coverage is important for contextualizing the applicability of our results. We have added discussion of this consideration to the Conclusion section.

“While high OH coverages require negative electrode potentials for thermodynamic stability (e.g., -0.75 V/SHE for 8/9 coverage at pH 0), moderate coverages (5/9 and 6/9) that are stable near typical HER operating conditions already provide substantial kinetic benefits with nearly barrierless Volmer steps.”

**Reviewer #3 (Remarks to the Author):**

This study provides a systematic investigation into the influence of surface terminations (O/OH) in $Ti_2C$ MXene on the interfacial hydrogen-bond network and hydrogen evolution reaction kinetics. The proposed concept of "hydrogen-bond network connectivity" offers an innovative extension beyond the conventional ΔGH descriptor. Nevertheless, several key aspects of the work require further substantiation to strengthen its conclusions.

1 . The total simulation duration of 10 ps is reported for the MD studies. However, whether this timescale is sufficient for the system to attain complete equilibrium remains inadequately justified.

Reply: We agree with the reviewer on the importance of sufficient MD timescales. Our equilibration protocol consists of multiple stages to ensure adequate equilibration: (1) initial 10 ps classical MD equilibration using the TIP3P force field for efficient structural relaxation, (2) subsequent 5 ps CP-AIMD equilibration in VASP to refine the interfacial structure under DFT accuracy and constant-potential conditions, and (3) 5 ps production runs for free energy calculations.

The adequacy of our equilibration protocol is demonstrated by analyzing the time evolution of the interfacial distance between the MXene surface and the first water layer during the CP-AIMD equilibration phase. As shown in Figure S5 (and representative example in Figure 7a for 4/9 OH coverage), the interfacial distance stabilizes within approximately 2 ps of the CP-AIMD equilibration and remains stable thereafter. The rapid stabilization indicates that the classical pre-equilibration successfully prepared reasonable initial configurations, and the subsequent CP-AIMD equilibration quickly converges to the DFT-level equilibrium structure.

The combination of classical and ab initio equilibration provides both computational efficiency and accuracy. The 10 ps TIP3P equilibration ensures that water molecules adopt reasonable liquid-phase configurations and removes any artifacts from initial structure generation. The subsequent 5 ps CP-AIMD equilibration then refines these configurations under the accurate DFT description and establishes the correct interfacial electronic structure under constant-potential conditions. This approach is well-established in computational electrochemistry for efficiently preparing equilibrated electrode/electrolyte interfaces.

The 5 ps production runs provide sufficient statistics for converged free energy gradients via the slow-growth approach, as evidenced by the smooth and physically reasonable free energy profiles obtained (Figures 3-5). The absence of significant noise or discontinuities in these profiles further confirms adequate sampling.

We have clarified this multi-stage equilibration protocol in the revised manuscript. While the detailed simulation procedure was originally described in the Supporting Information, we have now added explicit mention of the three-stage protocol to the Methods section of the main manuscript (Page 27) to make this information more readily accessible to readers.

2. The study employs a solvation model with four explicit water layers (approximately 30 molecules). It is important to assess whether this model size is sufficient to capture the long-range hydrogen-bond networks relevant to the proton transfer mechanism under investigation.

Reply: We thank the reviewer for this question regarding the adequacy of our explicit solvation model. Our model employs four water layers containing approximately 30 water molecules, which we demonstrate is sufficient to capture the relevant hydrogen-bond networks and interfacial phenomena governing HER kinetics.

To assess whether our model size adequately captures the hydrogen-bond networks relevant to proton transfer, we analyzed the radial distribution function (RDF) between the reacting hydrogen atoms (center of mass of the two H atoms involved in the reaction) and the oxygen atoms of water molecules. This analysis, performed for both transition states of the Heyrovsky step, reveals the spatial extent of water involvement in the reaction mechanism.

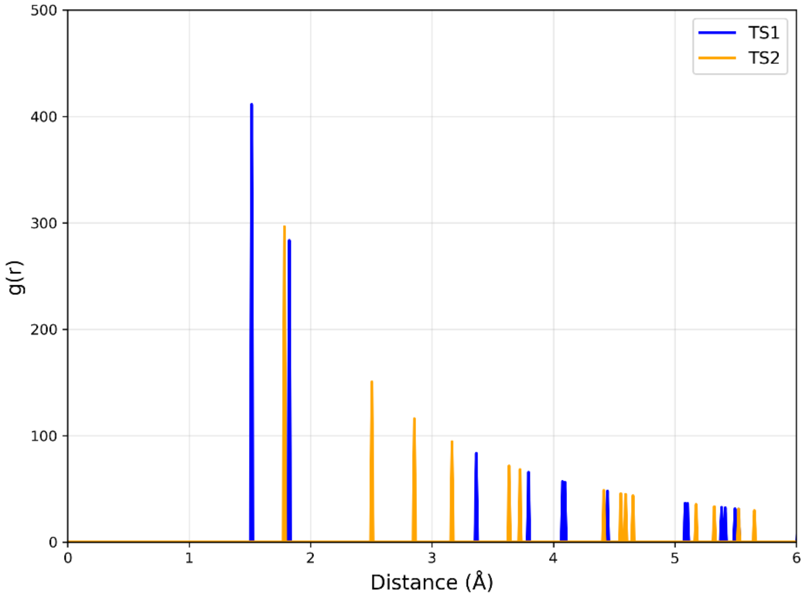


*Figure 6 Radial distribution functions g(r) calculated at the two transition states during the Heyrovsky step. The radial distribution function is calculated between the center of mass of the two reacting hydrogen atoms and the oxygen atoms of interfacial water molecules.*

The RDF shows a pronounced first solvation shell between 1-3 Å during the PCET transition state, with this peak largely diminishing by ~4 Å. By the second solvation shell (~5 Å), the RDF approaches bulk-like behavior, indicating that water molecules

beyond this distance do not significantly participate in the reaction mechanism. Given that our four-layer water model extends approximately 12 Å from the MXene surface, it fully encompasses the first and second solvation shells around the reactive species, ensuring complete representation of the hydrogen-bond networks directly involved in proton transfer.

During both Volmer and Heyrovsky steps, the reactions occur at the interface within the first 1-2 water layers, with the fourth layer exhibiting bulk-like behavior and minimal perturbation. The use of 3-4 water layers is well-established in computational electrochemistry for modeling electrode/electrolyte interfaces [*J. Am. Chem. Soc.*, 2023, 145, 25352; *Nat. Commun.*, 2024, 15, 10099]. The smooth free energy profiles and systematic coverage-dependent trends further validate that our model adequately represents the interfacial phenomena governing HER kinetics.

3 . The study assumes that the surface O/OH coverage remains constant during the reaction. However, under realistic electrocatalytic conditions, the electrode surface may undergo dynamic changes, such as the adsorption and desorption of OH species. It is necessary to discuss whether this assumption constitutes a limitation of the model.

Reply: We thank the reviewer for raising this point about surface dynamics. We would like to clarify our modeling approach and the rationale for treating surface O/OH coverage as constant during individual reaction events.

Our study investigates HER on $Ti_2C$ MXene under acidic conditions (pH = 0). The surface O/OH coverage at each electrode potential is determined from surface Pourbaix diagram analysis (Figure 2), which identifies the thermodynamically stable surface termination under given electrochemical conditions. For each coverage studied (1/9 to 8/9), we performed simulations at the corresponding electrode potential where that coverage is thermodynamically favorable. This approach ensures that our simulations reflect realistic surface compositions under operational conditions.

The assumption of constant surface coverage during individual HER reaction events is justified by significant timescale separation between elementary reaction steps and surface termination changes. Under steady-state HER operation at a fixed potential, the surface coverage reaches a quasi-equilibrium determined by the balance between hydroxylation and dehydroxylation rates, which is approximately what the surface Pourbaix diagram predicts thermodynamically.

Importantly, the surface OH groups defining "coverage" in our study are Ti-bonded spectator groups that enhance interfacial hydrogen bonding, not the reactive H* intermediates that form and desorb during the catalytic cycle. During HER, H atoms adsorb at surface O sites and subsequently desorb as $H_2$, regenerating the O site without altering the underlying O/OH termination pattern. This distinguishes local HER dynamics (which we simulate) from global surface composition changes (which occur on longer timescales).

Under realistic steady-state electrocatalytic conditions, the surface maintains an approximately constant average O/OH coverage determined by the applied potential and pH (as predicted by the Pourbaix diagram), even as individual HER turnovers continuously occur. While local fluctuations in coverage may arise, the overall surface composition remains stable over many catalytic cycles. Our approach of simulating HER kinetics at fixed, thermodynamically stable coverages therefore represents a reasonable model of steady-state catalytic operation.

We acknowledge that our model does not capture potential long-term surface evolution effects, such as gradual redistribution of O/OH terminations under prolonged polarization or kinetic trapping of metastable surface configurations. However, for understanding fundamental HER mechanisms and establishing structure-activity relationships between surface hydroxylation and catalytic performance, the assumption of constant surface coverage is both physically justified and necessary for computational tractability.

We have added relevant discussion to the Supporting Information under the section "CP-CMD simulations."

4. The physical-chemical origin of how OH terminations reduce the interfacial water-MXene separation remains unclear. Please provide further analysis to elucidate the fundamental mechanism behind this structural effect.

Reply: We thank the reviewer for the question about the fundamental mechanism behind the reduced interfacial separation with OH termination. The physical-chemical origin involves the complementary hydrogen bonding functionalities of mixed O/OH termination that enable better organization of the interfacial water structure.

The key mechanism is that surface O and OH groups provide complementary hydrogen bonding capabilities that work synergistically to organize the interfacial water layer. Surface O atoms (from O-termination) act as hydrogen bond acceptors, receiving H-bonds from water molecules. Surface OH groups (with H atoms pointing toward the aqueous phase) act as hydrogen bond donors, donating H-bonds to nearby water molecules. This combination of acceptor (O) and donor (OH) sites on the mixed-terminated surface creates a more diverse and favorable hydrogen bonding environment compared to pure O-termination, which provides only acceptor sites.

The complementary functionalities enable the surface to engage with water molecules in multiple binding geometries simultaneously, leading to enhanced overall hydrogen bond strength and network connectivity. Water molecules can optimize their orientations to accept H-bonds from surface OH groups while donating H-bonds to surface O sites, creating a more ordered and tightly bound first water layer.

Analysis of the interfacial distance as a function of OH coverage (Figure S5) reveals an important trend: the distance reduction becomes significant starting from 4/9 OH

coverage, with further increases in coverage providing diminishing improvements. This suggests that there is an optimal balance between O and OH functionalities for organizing the interfacial water structure. At intermediate coverages (around 4/9 to 6/9), the mixed O/OH termination provides sufficient diversity of binding sites to establish well-ordered water networks, while maintaining enough flexibility for water reorganization during proton transfer events.

At very low OH coverage (1/9 to 3/9), there are insufficient OH donor sites to fully organize the water layer, resulting in larger interfacial gaps. At very high coverage (7/9 to 8/9), the surface becomes dominated by OH groups with fewer O acceptor sites, which also limits the structural diversity. The optimal intermediate coverage provides the best balance of complementary functionalities.

This mechanism—where mixed surface functionalities with complementary hydrogen bonding capabilities enhance interfacial organization—aligns with prior studies of adsorbed hydroxyl species in electrocatalysis. For example, adsorbed OH species on Ru sites in Pt-Ru alloys similarly enhance H-bond connectivity and boost hydrogen electrocatalysis activity compared to pure Pt [Ref 14], suggesting that creating surfaces with diverse hydrogen bonding functionalities (both donors and acceptors) may be a general strategy for optimizing electrode/electrolyte interfaces.

We have added a brief discussion of this mechanism to the revised manuscript on Page 21.

5. The novelty of this work should be more clearly articulated through explicit comparison with existing theoretical studies, highlighting the distinct insights or advancements this study provides.

Reply: We agree that explicitly articulating our novelty through comparison with existing studies will strengthen the manuscript. Below we clarify the distinct insights and methodological advancements our work provides.

Our work shows the first application of constant-potential molecular dynamics to MXene HER with full PCET kinetics. The vast majority of theoretical studies on MXene HER rely on static DFT calculations using the computational hydrogen electrode (CHE) approach, with the Gibbs free energy of hydrogen adsorption ($\Delta G_H$) as the primary descriptor. Representative works include: i) metal doping studies showing that modified MXenes achieve $\Delta G_H$ values closer to the optimal zero (*Nat. Catal.*, 2018, 1, 985; *J. Am. Chem. Soc.*, 2019, 141, 17809), and ii) machine learning-aided descriptor discovery for MXene HER screening based on $\Delta G_H$ optimization (*J. Mater. Chem. A*, 2022,10, 18195; *J. Mater. Chem. A*, 2026, 14, 5349).

While these CHE-based studies successfully identify promising MXene compositions and establish thermodynamic trends, they fundamentally cannot capture: i) Dynamic interfacial water reorganization during proton transfer; ii) Potential-dependent surface

charge redistribution under realistic electrochemical conditions; iii) Kinetic barriers and actual reaction mechanisms beyond thermodynamic estimates. The CHE approach assumes an idealized proton transfer process without explicit treatment of the interfacial environment, which our work demonstrates is critical for understanding HER kinetics on MXenes.

Our work represents the first application of constant-potential constrained molecular dynamics (CP-CMD) simulations to investigate HER mechanisms on MXenes with explicit treatment of the electrified interface under grand canonical ensemble conditions. This enables us to directly compute activation barriers and capture the dynamic coupling between electrode potential, interfacial water structure, and proton transfer kinetics—aspects that are completely inaccessible to conventional CHE-based approaches.

Beyond the specific findings for $Ti_2C$ MXenes, our work establishes a computational framework for studying PCET processes on 2D materials that transcends the thermodynamic screening paradigm. The CP-CMD protocol we developed—integrating surface Pourbaix analysis and rigorous potential control—provides a template applicable to other MXene systems and 2D electrocatalysts more broadly. Our identification of interfacial optimization as a design principle distinct from binding energy tuning opens new avenues for rational catalyst design.

We have added the discussion of this novelty to the revised manuscript on Page 21.
